# Modulators of calcium signalling at fertilization

**DOI:** 10.1098/rsob.200118

**Published:** 2020-07-15

**Authors:** Paula Stein, Virginia Savy, Audrey M. Williams, Carmen J. Williams

**Affiliations:** 1 Reproductive and Developmental Biology Laboratory, National Institute of Environmental Health Sciences, National Institutes of Health, Research Triangle Park, NC 27709, USA; 2 Department of Molecular Genetics and Cell Biology, The University of Chicago, Chicago, IL 60637, USA

**Keywords:** fertilization, calcium signalling, calcium channels, egg activation, oocyte

## Abstract

Calcium (Ca^2+^) signals initiate egg activation across the animal kingdom and in at least some plants. These signals are crucial for the success of development and, in the case of mammals, health of the offspring. The mechanisms associated with fertilization that trigger these signals and the molecules that regulate their characteristic patterns vary widely. With few exceptions, a major contributor to fertilization-induced elevation in cytoplasmic Ca^2+^ is release from endoplasmic reticulum stores through the IP3 receptor. In some cases, Ca^2+^ influx from the extracellular space and/or release from alternative intracellular stores contribute to the rise in cytoplasmic Ca^2+^. Following the Ca^2+^ rise, the reuptake of Ca^2+^ into intracellular stores or efflux of Ca^2+^ out of the egg drive the return of cytoplasmic Ca^2+^ back to baseline levels. The molecular mediators of these Ca^2+^ fluxes in different organisms include Ca^2+^ release channels, uptake channels, exchangers and pumps. The functions of these mediators are regulated by their particular activating mechanisms but also by alterations in their expression and spatial organization. We discuss here the molecular basis for modulation of Ca^2+^ signalling at fertilization, highlighting differences across several animal phyla, and we mention key areas where questions remain.

## 1. Introduction

How the terminally differentiated gametes interact with each other at fertilization to initiate the development of a new organism is a question that has fascinated scientists for over a century. The earliest studies of fertilization, largely performed in marine animals, were summarized in 1919 by Frank Lillie in *Problems of Fertilization*. In this treatise, he states regarding fertilization:

It [fertilization] is the central decisive event in the genesis of all sexually produced animals and plants. Thus from one point of view it envisages the entire problem of sex; from another point of view it constitutes the basis of all development and inheritance. The elements that unite are single cells, each usually incapable, under natural conditions, of continued existence or development—on the point of death; but by their union a rejuvenated individual is formed which constitutes a link in the eternal procession of life by virtue of its power of reproduction. [1]

Despite this early scientific interest in fertilization, it was not until almost 60 years later that Lionel Jaffe and co-workers discovered that the sperm initiates a wave of calcium (Ca^2+^) across the egg (as initially proposed by Dalcq in 1928 [2]) and that this signal triggers the formation of a ‘rejuvenated individual’—the newly developing embryo [3,4]. These first experiments documenting that Ca^2+^ initiates embryonic development at fertilization were performed in eggs of medaka, a freshwater fish, and were made possible by the identification of jellyfish aequorin as a bioluminescent Ca^2+^-sensing protein [5]. Similar experiments performed shortly thereafter showed that Ca^2+^ was released following the fertilization of sea urchin eggs [6]. With the development of new chemical and genetically encoded Ca^2+^ sensors and advances in experimental techniques of *in vitro* fertilization (IVF) and Ca^2+^ imaging, we now know that a Ca^2+^ rise signals the initiation of animal development with no exceptions with cross-phyla sampling and even in several species of flowering plants [7,8]. However, the pattern of Ca^2+^ rises at fertilization varies widely. Fertilization-induced Ca^2+^ changes depend on a large number of variables including exactly how the Ca^2+^ rise is initiated and how the newly formed zygote responds to this Ca^2+^ rise and perhaps to other signalling pathways triggered at fertilization. The collective set of responses, including the Ca^2+^ rise, that is required for the oocyte to begin development into an embryo is termed ‘egg activation’. Here, we will review the generation and modulation of Ca^2+^ signals during egg activation, focusing mainly on knowledge gained from intensive studies of mammalian fertilization over the past 30 years. However, we will also draw from studies of non-mammalian animals to illustrate additional mechanisms of generating and modulating the Ca^2+^ signals that initiate embryonic development.

Ca^2+^ is a ubiquitous second messenger responsible for numerous cellular responses to external stimuli. Its ubiquitous nature implies that Ca^2+^ signals must be tightly regulated and that additional information must be encoded in the localization, amplitude and timing of Ca^2+^ release events. In this way, different stimuli can be properly interpreted by the cell and result in the necessary downstream responses. For example, basal cytoplasmic Ca^2+^ levels maintained by constitutive Ca^2+^ release from the endoplasmic reticulum (ER) suppress autophagy whereas higher levels of cytoplasmic Ca^2+^ promote autophagy [9]. In cardiac muscle cells, cytoplasmic Ca^2+^ signals promote contraction whereas nuclear Ca^2+^ pulses activate transcription of specific genes [10]. In mast cells, localized Ca^2+^ influx across the plasma membrane drives specific transcriptional responses not induced by global cytoplasmic Ca^2+^ rises [11]. Cells release and regulate Ca^2+^ under the control of a ‘Ca^2+^ signalling toolkit’ composed of Ca^2+^-mobilizing signals, channels that regulate Ca^2+^ influx into the cytoplasm or release from intracellular stores, and pumps and exchangers that remove Ca^2+^ from the cytoplasm [12]. In the next section, we will provide an overview of the major molecular mechanisms used by somatic cells as their toolkit to regulate cellular Ca^2+^ signals. (For more detailed descriptions of the Ca^2+^ signalling toolkit, see excellent recent reviews [12–15].) This will be followed by an in-depth review of how the Ca^2+^ signalling toolkit is used at fertilization to generate the signals that drive embryo development.

## 2. Modulators of Ca^2+^ signals and homeostasis—an overview

**Figure 1.**
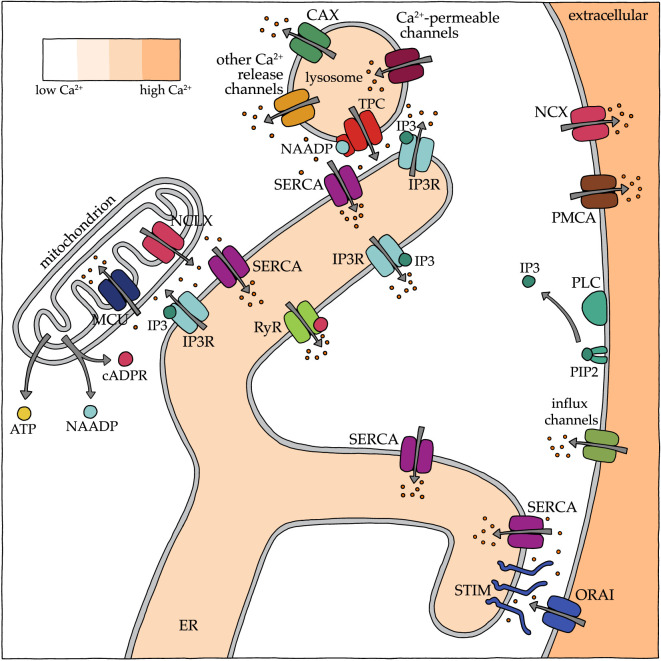
Elements of Ca^2+^ toolbox in endoplasmic reticulum, mitochondria, lysosomes and plasma membrane of somatic cells. Orange dots indicate Ca^2+^; grey arrows show direction of Ca^2+^ flow. cADPR, cyclic ADP ribose; CAX, Ca^2+^/proton exchanger; IP3, inositol trisphosphate; IP3R, IP3 receptor; MCU, mitochondrial uniporter; NCLX, mitochondrial sodium–Ca^2+^ exchanger; NAADP, nicotinic acid adenine dinucleotide phosphate; NCX, sodium/Ca^2+^ exchanger; ORAI, Ca^2+^ release-activated Ca^2+^ channel protein; PIP2, phosphatidylinositol 4,5-bisphosphate; PLC, phospholipase C; PMCA, plasma membrane Ca^2+^ ATPase; RyR, ryanodine receptor; SERCA, sarco/endoplasmic reticulum Ca^2+^ ATPase pump; STIM, stromal interaction molecule; TPC, two-pore channel.

Ca^2+^ signalling is feasible due to the presence of tightly regulated and localized Ca^2+^ gradients in the cell. A simplified schematic showing the relationships between the main modulators of cellular Ca^2+^ signalling is shown in figure 1. Ca^2+^ is available to cells from the extracellular environment, which has very high (approx. 2 mM) Ca^2+^ levels relative to cytoplasmic Ca^2+^ that is normally in the 100 nM range. The entry of extracellular Ca^2+^ into the cell is tightly regulated by Ca^2+^-permeable channels on the plasma membrane that are sensitive to a variety of stimuli. For example, voltage-gated Ca^2+^ channels open in response to changes in membrane potential, whereas the large family of transient receptor potential (TRP) channels responds to diverse stimuli including temperature, stretch and osmolarity [16,17]. ‘Store-operated Ca^2+^ entry’ (SOCE) is a cellular mechanism to replenish depleted ER Ca^2+^ stores with Ca^2+^ from the extracellular environment. When ER stores are depleted, ER-resident STIM proteins oligomerize and interact with ORAI plasma membrane Ca^2+^ channels to stimulate Ca^2+^ influx [18]. When open, all of these plasma membrane channels allow rapid entry of Ca^2+^ into the cytoplasm due to the large concentration gradient from extracellular to intracellular cytoplasmic compartments.

Intracellular Ca^2+^ is largely sequestered within the ER (or sarcoplasmic reticulum of muscle cells), but additional intracellular Ca^2+^ stores include endolysosomes and mitochondria. Peripheral tubular regions of the ER contain membrane contact sites with the plasma membrane and many organelles including lysosomes and mitochondria [19]. At these sites, apposing membranes do not merge but come into very close proximity, promoting the efficient transfer of high local concentrations of Ca^2+^ and other molecules between the two compartments. Ca^2+^ is released from ER stores mainly through two channels: the inositol-1,4,5-trisphosphate (IP3) receptor and the ryanodine receptor. Both of these channels are activated by Ca^2+^ itself, which can induce an autocatalytic process termed ‘Ca^2+^-induced Ca^2+^ release’ and lead to the propagation of waves of Ca^2+^ across a cell. IP3 and *sn*-1,2 diacylglycerol (DAG) are generated by the action of phosphoinositide-specific phospholipase C (PLC) enzymes on phosphatidyl inositol-4,5-bisphosphate (PIP2), often in response to ligand–receptor interactions at the plasma membrane. IP3 binding to IP3 receptors leads to Ca^2+^ release from ER stores in part by sensitizing the IP3 receptor to Ca^2+^. DAG can directly impact canonical TRP channel activity and indirectly influence Ca^2+^ signals by altering protein kinase C activity [20,21]. Two additional Ca^2+^ mobilizing signals, independent of IP3-mediated signalling, are generated from nicotinamide-adenine dinucleotide (NAD) and NADP: cyclic ADP ribose (cADPR) and nicotinic acid dinucleotide phosphate (NAADP) [22]. cADPR sensitizes the ER-associated ryanodine receptor to Ca^2+^, resulting in Ca^2+^ release from ER stores. NAADP, in contrast, signals to two-pore channels in lysosomes and other acidic organelles, resulting in Ca^2+^ release from those membranous stores. Mitochondria, which normally have relatively low Ca^2+^ stores, release Ca^2+^ through the sodium–Ca^2+^ exchanger, NCLX [23,24].

Once released into the cytoplasm, Ca^2+^ must be removed rapidly to terminate signalling and return cytoplasmic levels to baseline. Cytoplasmic Ca^2+^ removal is accomplished through the combination of extrusion from the cell and reuptake into intracellular stores. Extrusion across the plasma membrane is mediated by plasma membrane Ca^2+^ ATPase (PMCA) pumps and sodium–Ca^2+^ exchangers (NCX). Perhaps the most important mechanism of reuptake into intracellular stores is through sarco-ER Ca^2+^ ATPase (SERCA) pumps, which actively transport Ca^2+^ across a steep concentration gradient into the ER. Lysosomes probably also have Ca^2+^ uptake mechanisms, but the molecular basis for this function is not known [14]. Mitochondria take up Ca^2+^ through the mitochondrial Ca^2+^ uniporter and, under conditions of high cytoplasmic Ca^2+^, through the voltage-dependent anion channel [15]. Alterations in the expression, localization and activity of these modulators of Ca^2+^ signals determine the timing and localization of Ca^2+^ release, reuptake and extrusion. The end result is extraordinary variability in the final Ca^2+^ signals experienced by different regions of the cell, explaining the wide variety of distinct signalling pathways that use Ca^2+^ as a second messenger.

## 3. Preparation for Ca^2+^ signalling during oocyte maturation

In all animals, oocytes are arrested in prophase of meiosis I while they undergo growth and differentiation. In response to hormonal or developmental signals, oocytes undergo nuclear maturation, which entails completion of two rounds of chromosome segregation, and cytoplasmic maturation, which is a general term for the changes in cytoplasmic contents and organization necessary to support fertilization and embryo development. The oocyte's prophase I nucleus is termed the ‘germinal vesicle’ (GV) and we refer to oocytes in this meiotic stage as GV oocytes. Following the maturation signal, the GV breaks down (GVBD) and meiosis resumes. Subsequent stages of meiosis may continue without interruption, such as in *Caenorhabditis elegans* and some molluscs, or meiosis may arrest again at various stages to coordinate with the timing of fertilization [25]. In most insects, the second arrest is at metaphase I, whereas in most vertebrates the second arrest is at metaphase II. For the purposes of this review, we will refer to oocytes that have resumed meiosis and then secondarily arrested at another meiotic stage in preparation for fertilization as ‘eggs’.

Even though GV oocytes possess a Ca^2+^ signalling toolkit, their capacity to elicit Ca^2+^ responses is reduced compared with eggs, indicating that the ability to mount a robust Ca^2+^ response is acquired during oocyte maturation. This concept was first discovered in starfish oocytes, in which Ca^2+^ release in response to fertilization is far lower in immature oocytes relative to mature eggs, despite having similar Ca^2+^ stores [26]. In the mouse, GV oocytes fertilized *in vitro* display Ca^2+^ oscillations of lower amplitude, frequency and persistence than those observed in eggs [27–29]. Similarly, Ca^2+^ release in response to IP3 or PLC*ζ* injection is reduced in GV oocytes compared with eggs [28,30,31]. Several processes that take place during mouse oocyte maturation probably contribute to this difference in Ca^2+^ response: increase in Ca^2+^ stores, ER redistribution and increase in sensitivity to IP3, probably due to upregulation and phosphorylation of IP3 receptors. Ca^2+^ stores increase about four-fold during mouse meiotic maturation [29,32]. Interestingly, the smaller store size in GV oocytes appears to be due to a constant Ca^2+^ leak out of the ER through the IP3 receptor [33]. This leak ends around the time of GVBD, which is when the expansion of the ER Ca^2+^ store commences.

An increase in sensitivity to IP3 during oocyte maturation was first described in starfish oocytes and later demonstrated in hamster and mouse oocytes [26–28]. In the mouse, this finding is explained in part by a doubling of protein levels of the major IP3 receptor isoform, IP3R1, because inhibiting this maturation-associated increase in IP3R1 reduces IP3 sensitivity and alters Ca^2+^ oscillatory behaviour following fertilization [30,34–36]. During oocyte maturation, a redistribution of IP3R occurs, which mirrors ER redistribution (see below) in terms of localization and mechanisms [30,31,37]. However, altering IP3R1 redistribution using actin filament and microtubule depolymerizing agents does not change the sensitivity to IP3 [31]. As a general rule, IP3R1 phosphorylation enhances the conductivity of the channel. IP3R1 possesses phosphorylation consensus sites for numerous kinases, including M-phase kinases [such as polo-like kinase 1 (Plk1), mitogen-activated protein kinase (MAPK), cyclin-dependent kinase 1 (CDK1)], Ca^2+^/calmodulin-dependent protein kinase II (CaMKII), protein kinase A (PKA) and protein kinase C (PKC) [38]. Using the MPM-2 antibody, which recognizes an epitope present in proteins phosphorylated in the M-phase of the cell cycle, Lee *et al*. showed that MPM2 immunoreactivity of IP3R1 was barely detectable at the GV stage, increased sharply around GVBD, reached peak levels at MI and MII, and decreased again after fertilization [38]. This time-course of IP3R1 phosphorylation coincides with the activity of CDK1 and MAPK. Consistent with this finding, oocyte maturation is accompanied by an increase in IP3R1 phosphorylation at the CDK1 consensus sites S421 and T799 [31]. Furthermore, overexpression in eggs of a phosphomimetic IP3R1 protein in which the two CDK1 consensus sites (S421 and T799) and the ERK consensus site (S436) were mutated to aspartic acid led to increased sensitivity of the receptor to PLC*ζ* injection or caged IP3 expression [39]. These findings strongly suggest that CDK1- and MAPK-mediated phosphorylation of IP3R1 explains at least part of the increase in IP3 sensitivity that occurs during maturation. Phosphorylation of IP3R1 by PKA occurs in oocytes but this phosphorylation is maximal at the GV stage and decreases around GVBD, so it is unlikely to contribute to the increase in IP3 sensitivity with maturation [31]. Whether or not additional kinases and/or phosphatases impact IP3R1 sensitivity is unknown.

The IP3R is not the only Ca^2+^ regulatory protein whose amount increases during maturation and has an important function in eggs. Regulator of G-protein signalling 2 (RGS2) is a protein that blocks the activity of both G*α*s and G*α*_q_ proteins, thus inhibiting heterotrimeric G-protein-coupled receptor pathways that frequently trigger Ca^2+^ release. RGS2 protein levels are extremely low in GV oocytes, but the mRNA is recruited for translation during oocyte maturation, such that RGS2 levels increase approximately 20-fold [40]. RGS2 prevents premature Ca^2+^ release in eggs, which helps to prevent spontaneous egg activation events from beginning inappropriately prior to fertilization.

During the course of oocyte maturation, there is a reorganization of several organelles, including the ER, Golgi apparatus and mitochondria. Because the ER is the main cellular Ca^2+^ store, ER redistribution during oocyte maturation has been extensively studied and is correlated with the acquisition of a robust Ca^2+^ response. Using the fluorescent lipophilic dye DiI to label ER membranes, studies in starfish, marine nemertean worm, frog, hamster, human, mouse and plant oocytes demonstrated changes in ER organization during oocyte maturation [41–47]. In mouse, the ER is evenly distributed throughout the cytoplasm of GV oocytes with small accumulations in the interior, but not the cortical area, of the cell [46]. During GVBD, the ER becomes denser and envelops the meiotic spindle as it migrates towards the cortex [48]. The egg exhibits bright cortical clusters of ER, about 1–2 µm in diameter, that are absent from the region overlying the meiotic spindle [46,48]. The localization of both ER and IP3 receptors to cortical clusters in the egg situates the egg's main Ca^2+^ store and its releasing channel in proximity to the site of sperm–egg fusion, which is where the propagating Ca^2+^ wave starts at fertilization. The mechanism of this ER redistribution is a multi-step process driven by both microtubules and microfilaments [48]. Cytoplasmic lattices, unique to mammalian oocytes and preimplantation embryos, appear to have a role in ER redistribution during oocyte maturation because both *Padi6*- and *Nlrp5*-deficient oocytes, which lack these structures, fail to form cortical clusters [49,50]. Furthermore, Ca^2+^ oscillations are impaired in *Nlrp5* null eggs [50]. In *Xenopus* oocytes, the ER-rich structures found in immature oocytes have been characterized as annulate lamellae (AL). AL are rich in IP3 receptor content, but paradoxically show minimal Ca^2+^ release activity, as opposed to ER patches in eggs, from which most Ca^2+^ is released in response to IP3. This finding suggests that AL are a functional Ca^2+^ store with attenuated IP3 receptor activity [51]. Whether this structure plays a similar role in mammalian oocytes is unknown. Although the pattern of ER organization varies in different species, the fact that all species examined change this pattern during oocyte maturation suggests a conserved and functional role for ER redistribution to prepare the egg for fertilization [52].

The Golgi apparatus of mouse, rhesus monkey and bovine GV oocytes comprises a series of cytoplasmic stacks, or ‘mini-Golgis’, more abundant in the interior than in the cortical region of the cell [53,54]. Despite their unusual appearance, these mini-Golgis are perturbed by the membrane trafficking inhibitor brefeldin A, indicating that they are functional. This experiment also demonstrated a role for membrane trafficking during oocyte maturation because brefeldin A-treated oocytes fail to complete meiosis I and arrest after GVBD [53]. During GVBD, these mini-Golgis fragment and become dispersed more homogeneously throughout the cytoplasm for the remainder of maturation. This pattern is reminiscent of what takes place in somatic cells entering mitosis [55]. The Golgi apparatus is estimated to provide about 5% of the total cellular Ca^2+^ store. Golgi membranes contain functional IP3 receptors [56], but the possible contributions of this organelle to Ca^2+^ release during egg activation is unknown. It is also unclear if there is an important role for the reorganization of the Golgi apparatus during oocyte maturation, or whether it is simply a response to major ER rearrangements or changing trafficking demands. One possible role for the Golgi fragments is serving as an intracellular source of PIP2 for hydrolysis by PLC*ζ* at fertilization. The amount of vesicular PIP2 increases during oocyte maturation and, interestingly, the localization pattern of both PIP2 and PLC*ζ* suggests that the vesicles could be part of the Golgi apparatus [57].

Mitochondria also undergo redistribution during oocyte maturation. At GVBD, they form a dense ring in the perinuclear area, followed by dispersion throughout the cytoplasm and formation of a new mitochondrial ring around the meiotic spindle [58–60]. By the metaphase II stage, mitochondria become homogeneously distributed throughout the cytoplasm, with very few localized in the polar body [59]. There is also a change in mitochondrial density or clustering during oocyte maturation that correlates with changes in ATP production (i.e. ATP production is high when mitochondria are found in large clusters [60]). Because mitochondria are essential for sustaining Ca^2+^ oscillations, at least in part by providing the ATP required for Ca^2+^ pumps, these changes in mitochondrial localization, clustering and ATP output during oocyte maturation may be necessary to elicit a robust Ca^2+^ response at fertilization.

## 4. Shapes and consequences of fertilization-associated Ca^2+^ signals

**Figure 2.**
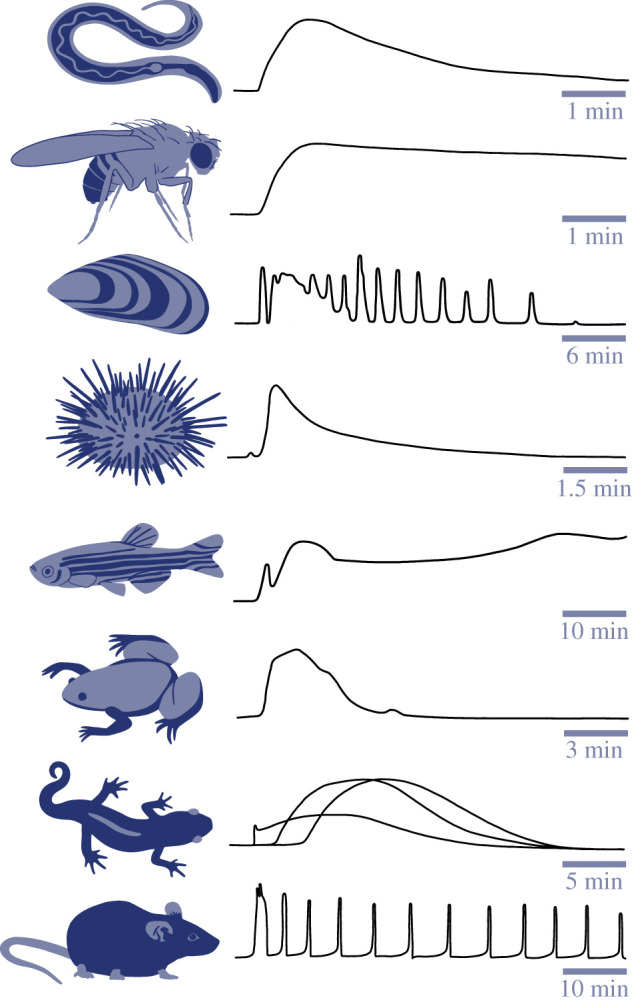
Diagrams of typical cytoplasmic Ca^2+^ changes at fertilization across various species. From top to bottom: nematode (*Caenorhabditis elegans*) [62], fruit fly (*Drosophila melanogaster*) [63], purple mussel (*Septifer virgatus*) [7], sea urchin (*Paracentrotus lividus*) [64], zebrafish (*Danio rerio*) [65], frog (*Xenopus laevis*) [66], newt (*Cynops pyrrhogaster*) [67] and mouse (*Mus musculus*) [68]. Time indicated on scale bar for each Ca^2+^ trace. The three lines on the newt trace represent spatially distinct signals induced by different sperm during physiological polyspermy. Traces adapted from indicated references.

As mentioned previously, Ca^2+^ signalling is a universal hallmark of egg activation in all sexually reproducing species studied thus far. However, sperm-induced Ca^2+^ signals are significantly different across multiple phyla [61]. The diversity of Ca^2+^ dynamics during egg activation is schematized in figure 2. In most non-mammalian species, the fertilization-induced Ca^2+^ pattern takes the form of a single or a few waves. One exception is the marine worm *Urechis caupo*, in which the sperm triggers entry of extracellular Ca^2+^ uniformly around the egg cortex and no Ca^2+^ waves occur [69]. In medaka, sea urchins, frogs and *C. elegans*, a local cytoplasmic Ca^2+^ increase occurs immediately after sperm–egg fusion, and a single Ca^2+^ wave propagates from the sperm entry site across the entire egg [3,62,64,66,70]. The Ca^2+^ wave generally lasts from one to several minutes. *Drosophila* eggs also have a single Ca^2+^ wave; however, it is initiated from the egg pole through mechanical pressure and moves towards the centre of the oocyte and finally to the entire egg [63]. In species that undergo physiological polyspermy, such as newts and domestic fowl, each of the fertilizing sperm evokes a single slow Ca^2+^ wave that spreads across a small portion of the surface of the egg. Collectively, the multiple Ca^2+^ waves triggered around the whole egg ensure a global and long-lasting intracellular Ca^2+^ increase necessary for egg activation [71,72]. Another type of fertilization-induced Ca^2+^ pattern is a series of oscillations that travel in a wave-like fashion across the egg and last for minutes to hours. For example, fertilized marine nemertean worm and marine bivalve eggs display multiple oscillations that can persist for 30–60 min [73–75]. Similarly, mammalian eggs display a series of Ca^2+^ oscillations after sperm–egg fusion, but these persist for several hours [76,77].

Although Ca^2+^ dynamics vary widely among species, the Ca^2+^ oscillatory pattern is stereotypical for mammals. Shortly after sperm–egg fusion, the egg intracellular Ca^2+^ concentration rises more than 10-fold and persists close to this level for several minutes before returning to baseline. This initial relatively long Ca^2+^ transient is considered the main Ca^2+^ signal responsible for egg activation. The characteristic shape of this first Ca^2+^ transient has been well documented in different mammalian species using ratiometric Ca^2+^ imaging with epifluorescence signal detection [76,78–80]. Ca^2+^ concentration rises slowly at first and then rises rapidly to a peak, frequently followed by several short low-amplitude increases from the peak. The Ca^2+^ level then gradually falls over the course of several minutes before dropping rapidly back to baseline as cytoplasmic Ca^2+^ is cleared. The first transient is followed by a series of much shorter Ca^2+^ transients that are also characterized by an initial slow rise in Ca^2+^, a rapid rise to a peak that is usually lower than that of the first transient, and then rapid return to baseline [81]. In mammals, sperm-induced Ca^2+^ oscillations persist for several hours, until around the time of pronucleus formation. The frequency of Ca^2+^ oscillations appears to be a species-specific characteristic; however, it is possible that differences in media composition rather than inherent species differences influence oscillation frequency.

Long-lasting fertilization-associated changes in Ca^2+^ homeostasis orchestrate early and late events of egg activation. Artificial modulation of the Ca^2+^ oscillatory pattern by electropermeabilization revealed a correlation between the total number of Ca^2+^ transients applied and distinct cellular events of mouse egg activation [82]. Thus, cortical granule exocytosis is triggered by a single Ca^2+^ transient, whereas more than four transients are necessary to induce cell cycle resumption and polar body emission. Furthermore, robust pronuclear development, which is a later event of egg activation, requires additional electropermeabilization-induced Ca^2+^ transients. Studies in rabbit and mouse eggs concluded that the different egg activation events rely on a cumulative effect of Ca^2+^ input over downstream effectors, rather than a specific number or frequency of Ca^2+^ signals [83–85]. Therefore, eggs seem to be flexible regarding the Ca^2+^ pattern for activation, if the total summation of Ca^2+^ stimuli is sufficient to trigger egg activation.

Interestingly, experimental manipulation of Ca^2+^ signalling during or immediately after fertilization of mouse eggs results in alterations in the pre- and post-implantation developmental programs and even postnatal growth rate. (Note that the molecular mechanisms immediately downstream of Ca^2+^ signalling that promote egg activation are outside the scope of this review; for more information on this topic see [86,87].) In the mouse, experimental Ca^2+^ modulation following IVF causes alterations in the blastocyst transcriptome [84]. This finding is similar to observations in somatic cells, in which experimental modulation of Ca^2+^ signalling causes alterations in gene expression patterns [88]. Moreover, premature termination of the Ca^2+^ oscillatory pattern following fertilization results in lower implantation rates but no subsequent differences in development to term. By contrast, hyperstimulation of Ca^2+^ signalling following fertilization does not affect implantation but impairs development to term [84]. Similar experiments using rabbit eggs demonstrated that artificial modulation of Ca^2+^ dynamics during egg activation significantly affects implantation rates [83]. It is notable that the modulation of Ca^2+^ signalling during the relatively short time of egg activation also has long-term effects on offspring growth. Mouse offspring derived from hyperstimulated eggs gained less weight after weaning and the variation in body weight was much greater than that of controls [84]. These findings regarding abnormal growth trajectory and weight variance were also observed in two different mouse models of decreased Ca^2+^ signalling following fertilization *in vivo* [89]. Therefore, fine regulation of Ca^2+^ signalling following fertilization is critical to obtain developmentally competent embryos and healthy offspring. A link between abnormal patterns of Ca^2+^ signalling after IVF and the long-term effects on growth trajectory could be the impact of Ca^2+^ signalling on egg redox state and mitochondrial activity [90].

Despite advances in our understanding, it remains unclear whether these findings reported mainly from *in vitro* studies recapitulate the complex regulation of *in vivo* fertilization, in terms of the need for a specific Ca^2+^ oscillatory pattern for developmental success. Doubts arose after it was reported that several different genetic mouse models in which eggs show a very abnormal pattern of sperm-induced Ca^2+^ oscillations after IVF were still capable of producing offspring when fertilization occurred *in vivo* [89,91,92]. These reports suggest that there may not be a need for a prolonged series of Ca^2+^ oscillations to activate eggs effectively *in vivo*. Further studies are needed to define the fertilization-associated Ca^2+^ oscillatory patterns *in vivo*, within the highly specialized environment of the oviduct, and to determine whether alterations in Ca^2+^ signalling *in vivo* have similar detrimental effects on offspring health as artificial disruption *in vitro*.

## 5. Modulators of Ca^2+^ signals at fertilization

### 5.1. Ca^2+^-mobilizing signals

IP3 is the major Ca^2+^ mobilizing signal at fertilization and was the first identified. Early biochemical studies in sea urchins documented a rapid rise in both triphosphoinositides and diphosphoinositides that occurred after insemination but prior to evidence of Ca^2+^ release, suggesting that IP3 mediates Ca^2+^ release as had been shown previously in somatic cells [93]. This idea was tested by microinjection of IP3 into sea urchin eggs, which resulted in exocytosis of cortical granules and elevation of the fertilization envelope, both indicators of an increase in cytoplasmic Ca^2+^ concentration [94]. Subsequent studies in frog, hamster, mouse, marine worms and tunicates documented that IP3-induced Ca^2+^ release occurs at fertilization across multiple phyla [95–98].

Besides IP3-induced Ca^2+^ release, some species use alternative or additional mechanisms. In sea urchins, cADPR serves as a redundant mechanism for Ca^2+^ release at fertilization, presumably by activating the ryanodine receptor [70,99]. There is also evidence in sea urchin and starfish that NAADP stimulates Ca^2+^ release at fertilization [100–102]. Ca^2+^ entry from the extracellular milieu serves as a mechanism to increase cytoplasmic Ca^2+^ in echinoderms, molluscs and worms. In starfish, Ca^2+^ entry is a response to sperm-induced activation of a voltage-gated Ca^2+^ channel; this interaction results in a ‘cortical flash’ of Ca^2+^ [103,104]. In sea urchins, both voltage-gated channels and NAADP-induced Ca^2+^ release contribute to the cortical flash [105]. Limpets are exceptional in that they depend exclusively on Ca^2+^ influx, and not intracellular stores, to provide the fertilization Ca^2+^ signal [106]. In *C. elegans*, Ca^2+^ enters the egg through TRP channels located in the sperm plasma membrane following sperm–egg fusion [62]. In this way, the sperm membrane becomes a ‘conduit’ for Ca^2+^ entry as was first proposed by Jaffe [107]. This mode of Ca^2+^ entry is responsible for the rapid local rise in Ca^2+^ that precedes the global wave that crosses the egg, analogous to the cortical flash in starfish, but is not essential for the occurrence of the later global Ca^2+^ wave or for embryogenesis.

Some species undergo physiological egg activation in the absence of sperm. For example, the Ca^2+^ wave responsible for activating *Drosophila* eggs is initiated from extracellular Ca^2+^ sources in response to the mechanical stimulation of egg plasma membrane TRP channels during ovulation [63,108]. The propagation of the wave, however, depends on IP3 receptor-mediated Ca^2+^ release [108]. Similarly, spawning alone in species such as zebrafish and *Sicyonia* shrimp induces a Ca^2+^ wave in the absence of sperm [109,110]. In shrimp, the Ca^2+^ wave is initiated downstream of magnesium ions in seawater, but the molecular mechanism underlying Ca^2+^ entry is unknown [110]. Of note, separation of the initiation of the Ca^2+^ wave from the fertilizing sperm creates a requirement for highly efficient single sperm entry soon after egg activation to ensure that diploid embryonic development begins synchronously.

### 5.2. Phosphoinositide-specific phospholipase C

Generation of IP3 by PLC-mediated hydrolysis of PIP2 is essential for Ca^2+^ release at fertilization in most species studied, but the specific PLC used varies. There are six families of PLC enzymes in animals: PLC*β*, PLC*γ*, PLC*δ*, PLC*ε*, PLC*ζ* and PLC*η* [111]. Each PLC family is activated in distinct ways, but all PLC isoforms have a conserved catalytic domain and Ca^2+^-binding EF hand motifs. Hence, they all carry out PIP2 hydrolysis to generate IP3 and DAG and can be modulated by changes in Ca^2+^ levels.

The first experiments to identify a specific PLC that was activated at fertilization to generate the IP3 responsible for Ca^2+^ release were performed in the starfish *Asterina miniata*. Carroll and colleagues demonstrated that src homology-2 (SH2) domain-mediated inhibition of PLC*γ* activity prevents the sperm-induced Ca^2+^ wave that follows the cortical flash [112]. Similar experiments performed in the ascidian *Ciona intestinalis* revealed that PLC*γ* mediates Ca^2+^ release in these marine invertebrates as well [113]. These findings provided support for the idea that IP3 is the major downstream mediator of the Ca^2+^ wave. In addition, these findings suggested that a tyrosine kinase functions upstream of PLC*γ* activity at fertilization given that this is the most common mechanism of activating PLC*γ* [111]. Further studies in sea urchin and *Xenopus* demonstrated that src family tyrosine kinases stimulate Ca^2+^ release at fertilization by activating PLC*γ* [66,114]. Hence, a soluble tyrosine kinase/PLC*γ*/IP3 pathway to initiate Ca^2+^ release appears to be a common mechanism among external fertilizing species. However, in mouse, SH2-domain-mediated inhibition of PLC*γ* activity has no impact on sperm-induced Ca^2+^ oscillations, indicating that either PLC*γ* is not involved or that it must be activated by an alternative mechanism [115].

The hypothesis that sperm introduce a PLC activity into the egg that is responsible for PIP2 hydrolysis and Ca^2+^ release following sperm–egg plasma membrane fusion at fertilization was first proposed by Whitaker & Irvine [94]. This idea was based on studies in sea urchins presented earlier that year demonstrating that injection of sperm extracts results in the formation of the fertilization envelope, which occurs downstream of Ca^2+^-induced exocytosis of cortical granules [116]. Similar experiments using sperm extract injections in ascidian, rabbit, mouse and hamster eggs demonstrated that a factor present in sperm extracts could generate Ca^2+^-dependent egg activation events in distinct phyla [117–119]. Consistent with the idea that a soluble sperm factor could be a common physiological inducer of Ca^2+^ release at fertilization, sperm–egg fusion precedes the increase in cytoplasmic Ca^2+^ in sea urchin, frog and mouse [95,120–122]. However, it was not until 2002 that a novel sperm-specific PLC, PLC*ζ*, was cloned from a mouse spermatid cDNA library and identified convincingly as the protein in sperm extracts capable of activating eggs [123]. Microinjection of cRNA encoding PLC*ζ* caused Ca^2+^ oscillatory patterns very similar to those observed at fertilization. Importantly, removal of PLC*ζ* from hamster sperm extracts by an immunodepletion strategy caused the loss of the extract's Ca^2+^ releasing activity following microinjection into mouse eggs. Finally, mutation of a critical catalytic site residue abrogated this activity, indicating that it was the PLC*ζ* enzymatic activity that was responsible for Ca^2+^ release. These studies effectively demonstrated that PLC*ζ* was the critical component in microinjected sperm extracts that induces Ca^2+^ release, but left open the question of whether or not PLC*ζ* was important for fertilization-induced Ca^2+^ release.

The first *in vivo* model to describe a role for PLC*ζ* at fertilization was generated using a mouse transgenic RNA interference (RNAi) knockdown approach that resulted in an approximately 40% reduction in sperm PLC*ζ* protein levels [124]. Eggs fertilized *in vitro* by these sperm had abnormal patterns of Ca^2+^ oscillations that terminated prematurely. Subsequent experiments using human sperm lacking PLC*ζ* obtained from infertile males demonstrated that the sperm failed to induce Ca^2+^ release when injected into mouse eggs [125]. This finding was consistent with the clinical failure of human egg activation following intracytoplasmic sperm injection (ICSI) observed in these patients. Later, two different point mutations in the catalytic region of human *PLCZ1* were identified that were associated with a case of male infertility refractory to ICSI [126,127]. Both of these mutations were associated with deficient Ca^2+^ oscillation-inducing ability in mouse eggs [127,128]. Additional novel *PLCZ1* mutations associated with human egg activation failure have since been identified [129–132]. The recent development of three different mutant mouse *Plcz1* models confirmed that PLC*ζ* is essential for normal Ca^2+^ oscillations following fertilization [91,92]. Surprisingly, in all three mutant models, the males were not sterile but instead had very small litters. Both failure of egg activation (failure to form pronuclei) and polyspermy contributed to the subfertility. Careful evaluation of the Ca^2+^ oscillation phenotype in one of these models revealed that eggs fertilized *in vitro* by a single sperm exhibited approximately 3 Ca^2+^ transients on average, but that the onset of these transients was delayed by approximately 45–60 min relative to eggs fertilized with wild-type sperm [92]. Interestingly, sperm lacking PLC*ζ* did not induce any Ca^2+^ transients following ICSI, suggesting that events associated with sperm–egg plasma membrane fusion were essential for this PLCζ-independent Ca^2+^-releasing activity. The molecular basis for this additional egg activation mechanism is unknown.

PLC*ζ*, the smallest PLC identified to date, is unique when compared with previously identified PLCs [123]. It lacks a pleckstrin homology (PH) domain present in all other PLCs and lacks coiled coil, src homology, ras-association and RAS-GEF domains that are present in some but not all other PLCs [111]. Because PH domains mediate the association of PLCs with the plasma membrane through an interaction with their substrate PIP2, the lack of this domain in PLC*ζ* explains its ability to diffuse freely through the egg cytoplasm. Indeed, there is evidence that plasma membrane PIP2 does not serve as a substrate for PLC*ζ* at fertilization [57,133]. In mouse eggs, plasma membrane PIP2 levels do not decrease at fertilization. Additional PIP2 is localized to intracellular vesicles, and depletion of intracellular vesicle-associated PIP2 impairs Ca^2+^ oscillations induced by the fertilizing sperm [57,134]. The nature of the PIP2-associated vesicles is not known, but they are distinct from ER clusters. The interaction of PLC*ζ* with these vesicles appears to be mediated by basic residues in the XY-linker, EF-hand and C2 domains [129,135–137]. The catalytic activity of most PLC isoforms is autoinhibited by negatively charged residues in the XY-linker region; the enzymes are activated by various mechanisms of releasing autoinhibition [111]. This is not the case for PLC*ζ*. The XY-linker in PLC*ζ* is positively charged and its deletion causes a loss of enzymatic activity, suggesting that PLC*ζ* is constitutively active [138]. Ca^2+^ is the only known activator of PLC*ζ*, with an EC50 of approximately 50 nM and 70% maximal activity at 100 nM [139]. Hence, PLC*ζ* is almost maximally active at the basal intracellular Ca^2+^ levels present in the egg cytoplasm. By contrast, other PLCs are activated by approximately 10-fold higher Ca^2+^ levels [111].

Although PLC*ζ* is responsible for the PLC activity that initiates Ca^2+^ release during egg activation, an open question is whether or not endogenous egg PLCs contribute to the Ca^2+^ oscillatory pattern by generating additional IP3-mediated Ca^2+^ release. On somatic cell membranes, ligand-G-protein-coupled receptor interactions frequently stimulate PLC*β* isoform-mediated generation of IP3 and DAG through the activity of heterotrimeric G-protein *α* subunits in the G*α*_q_ family [111]. By analogy, one proposed mechanism for sperm-mediated egg activation was through a sperm ligand–egg receptor interaction that could activate PLC*β*. Support for this mechanism came from the observation that overexpression of the G*α*_q_-coupled m1 muscarinic receptor in mouse eggs followed by exposure to its cognate ligand, acetylcholine, caused egg activation events downstream of cytoplasmic Ca^2+^ elevation [140,141]. However, subsequent experiments demonstrated that although stimulation of G*α*_q_ could cause complete egg activation, sperm did not require this activity to successfully activate eggs, indicating that G*α*_q_-mediated PLC*β* stimulation was not required for Ca^2+^ release at fertilization [142]. The role of PLC*β* was also tested using a knockdown strategy to generate mice carrying eggs with decreased levels of PLC*β*1 [143]. Reduction in PLC*β*1 caused a significant decrease in the amplitude of sperm-induced Ca^2+^ transients, but did not impact other characteristics of the oscillatory pattern, suggesting a contributory but not major role for PLC*β*1. An interesting recent study took advantage of the development of a new fluorescent IP3 sensor to monitor both Ca^2+^ and IP3 concurrently in fertilized mouse eggs [144]. Using this tool, it was shown that during later phases of the Ca^2+^ oscillations following fertilization, Ca^2+^ activates PLC to generate IP3 peaks that follow each Ca^2+^ transient. These findings further support the idea that endogenous egg PLCs are active at fertilization and impact the overall Ca^2+^ signals experienced by the early embryo, but which PLC, if any, has yet to be determined.

### 5.3. IP3 receptor

**Figure 3.**
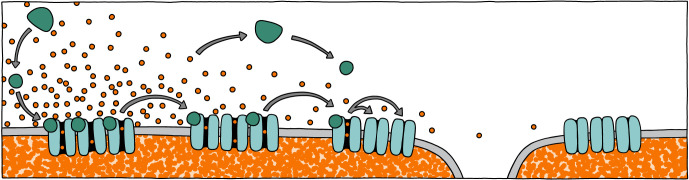
Generation of a Ca^2+^ wave. Localized production of IP3 (teal circles) by PLC (teal semicircles) leads to IP3-mediated Ca^2+^ release from clusters of IP3 receptors in endoplasmic reticulum (ER) membranes. The released Ca^2+^ promotes Ca^2+^-induced Ca^2+^ release from nearby clusters, increasing the cytoplasmic Ca^2+^ gradient. In addition, the released Ca^2+^ stimulates PLC to generate additional IP3 in a positive feedback loop. Continued IP3 production and Ca^2+^ diffusion lead eventually to Ca^2+^ release from distal IP3 receptor clusters in distinct ER regions and a wave of Ca^2+^ release across the cell. Orange dots indicate Ca^2+^; grey arrows show the direction of IP3 or Ca^2+^ flow.

The IP3 receptor is an ion channel that releases Ca^2+^ from the ER in response to IP3 and Ca^2+^ binding. It is composed of large subunits that assemble as tetramers to generate a greater than 1 MDa Ca^2+^ channel mainly present in ER membranes. Only a small portion of the IP3 receptor extends into the ER lumen; the majority extends into the cytoplasm, giving it a square mushroom-shaped structure that is evident by cryo-electron microscopy [145]. Each subunit has a single IP3-binding site. IP3 receptor opening requires binding of IP3 to each of the four subunits along with Ca^2+^ binding, which has a biphasic impact on channel opening [146–148]. Small increases in cytoplasmic Ca^2+^ levels promote channel opening, whereas high (greater than 300 nM) Ca^2+^ concentrations inhibit channel opening. Hence, Ca^2+^ released by channel opening feeds back on the IP3 receptor to promote closing, resulting in intermittent Ca^2+^ release events from single receptors. When IP3 receptors are enriched in specific locations, this feedback system leads to localized Ca^2+^-induced Ca^2+^ release as nearby receptors open in response to Ca^2+^ released by others. Finally, as sufficient Ca^2+^ is released to enable diffusion to more distant sites, distal IP3 receptors open. When supported by Ca^2+^-induced activation of PLC to generate additional IP3, this process can eventually lead to the formation of regenerative Ca^2+^ waves that travel across the cell (figure 3) [94,134,149]. Generation of these Ca^2+^ waves depends on the absolute number, spacing and localization of the IP3 receptors as well as the localization and intensity of the signals generating the necessary IP3 and Ca^2+^ inputs.

The large cytoplasmic portion of the receptor supports the modulation of IP3 receptor function by numerous agonists and antagonists in addition to IP3 and Ca^2+^ (reviewed in [150,151]). These modulators include other small molecules such as ATP and NADH, reactive oxygen species, and Ca^2+^-binding proteins such as calmodulin. IP3 receptor opening is also modulated by phosphorylation/dephosphorylation and other covalent modifications including ubiquitination, cross-linking and limited proteolysis. For example, PKA, PKC and CaMKII all phosphorylate the IP3 receptor to regulate channel opening [152–154]. Hence, the IP3 receptor serves as a signalling hub that integrates inputs from many different signalling pathways to drive the resulting Ca^2+^ signals received by the cell.

The release of Ca^2+^ through the IP3 receptor is essential at fertilization for most animals studied. This was first demonstrated in hamster eggs, in which microinjection of a function-blocking monoclonal antibody that inhibits Ca^2+^ release from the IP3 receptor prevented sperm-induced Ca^2+^ oscillations following fertilization [155]. Similar studies in mouse eggs demonstrated that the antibody blockade of IP3 receptor function completely prevented egg activation [156]. In starfish, the critical role of IP3-receptor-mediated Ca^2+^ release at fertilization was demonstrated using an ‘IP3 sponge’ that prevents receptor activation by absorbing IP3 [37]. Although IP3 receptor activity is essential for sea urchin fertilization, the release of Ca^2+^ through the related ER-resident ryanodine receptor is also necessary for a full Ca^2+^ release response [99,104]. In ascidians, the IP3 receptor generates Ca^2+^ release responsible for the fertilization-associated Ca^2+^ wave, but Ca^2+^ released through the ryanodine receptor promotes post-fertilization plasma membrane insertion events that do not occur when only the IP3 receptor is active [157]. Although the ryanodine receptor can be detected in mammalian eggs, it does not appear to contribute significantly to Ca^2+^ release or other egg activation events at fertilization in mammals [158–161].

Many animals, including *Xenopus*, *Drosophila*, *C. elegans* and starfish, appear to have a single IP3 receptor isoform [37,162–164]. However, three highly homologous IP3 receptor subunits (IP3R1, IP3R2 and IP3R3) are found in mammals; several splice variants also have been identified [165]. The IP3 receptor can be composed of either homotetramers or heterotetramers of distinct subunit types [166]. Although all subunit types carry out similar Ca^2+^ release functions in response to IP3 and Ca^2+^, there are differences in their regulatory properties. For example, IP3R2 has a higher affinity for IP3 than IP3R1, and IP3R1 and IP3R2 are activated when phosphorylated by PKA, but IP3R3 is not [167–169]. IP3R1 has been detected in mouse and bovine eggs where it is enriched in the cortical region [30,170]. In mouse and bovine eggs, IP3R2 and IP3R3 also can be detected; however, IP3R1 is far more abundant [34,36,170]. Hence, it is likely that IP3R1 carries out the majority of the fertilization-induced Ca^2+^ release activity in mammalian eggs as first demonstrated in hamster eggs by Miyazaki *et al.* [155].

An important consideration regarding the regulation of IP3 receptor activity is the particular ER subdomains where the receptor is localized. As mentioned above, IP3 receptor quantity, localization and phosphorylation status change during oocyte maturation in mammals and these changes probably impact Ca^2+^ signalling. In *Xenopus* oocytes, IP3 receptor quantity changes minimally during maturation but there are still significant differences in IP3 receptor-mediated Ca^2+^ release events between oocytes and eggs [51,163]. It turns out that these differences are modulated in part by the association of IP3 receptors with distinct ER subdomains such as annulate lamellae, which are remodelled during oocyte maturation [51]. In mammalian eggs, specific differences in IP3 receptor behaviour based on localization to ER subdomains have not yet been demonstrated.

### 5.4. Two-pore channels

Two-pore channels, which localize to acidic organelles of the endolysosomal system, release Ca^2+^ in response to stimulation by NAADP. These channels were first identified based on their similarities to voltage-gated Ca^2+^ channels and TRP channels [171]. Their identification provided a possible molecular basis for the observation originally made in sea urchin egg homogenates that NAADP releases Ca^2+^ from reserve (yolk) granules, a cellular compartment distinct from the ER and a functional equivalent of lysosomes [172]. The reserve granules mediate the contribution of NAADP to the generation of the cortical flash during sea urchin fertilization, probably through one of the sea urchin TPC isoforms, though a direct connection has not yet been documented [105]. More recent studies indicate that NAADP interacts with an accessory protein that modulates TPC opening rather than directly with the TPC [173]. It is likely that only small amounts of Ca^2+^ are released from endolysosomal stores in response to NAADP; however, these Ca^2+^ release events can then trigger Ca^2+^-induced Ca^2+^ release mediated by ER-associated IP3 and/or ryanodine receptors [22,174]. Likewise, IP3 receptor-mediated Ca^2+^ release from ER stores can promote Ca^2+^ release from acidic stores through retrograde signalling [175]. This crosstalk appears to depend on the close apposition of ER and acidic stores at membrane contact sites [19,176].

### 5.5. Sarco/endoplamic reticulum Ca^2+^-ATPase pumps

The sarco-ER Ca^2+^-ATPase (SERCA) is a P-type Ca^2+^ pump located in ER membranes that transports Ca^2+^ against a concentration gradient from the cytoplasm into the ER lumen. In mammals, it is encoded by three genes (*Atp2a1–3*) that generate the three main isoforms (SERCA1–3), but because the transcripts are subject to alternative splicing, they give rise to 11 SERCA isoforms [177]. These isoforms differ in their tissue and developmental expression patterns, affinity for Ca^2+^, and regulation. SERCA1a and 1b are expressed in adult and foetal fast-twitch skeletal muscle, respectively. SERCA2a is expressed in cardiac and slow-twitch skeletal muscle, whereas SERCA2b is ubiquitously expressed and considered the housekeeping SERCA isoform. SERCA3 isoforms are found in certain non-muscle cells, usually co-expressed with SERCA2b. The functional organization of SERCA proteins, like all Ca^2+^-ATPases, is composed of a transmembrane domain containing 10 membrane-spanning helices and three cytoplasmic domains: the actuator, phosphorylation and nucleotide-binding domains. The SERCA pump contains two Ca^2+^-binding sites in the transmembrane domain and hence transports two Ca^2+^ ions per ATP hydrolyzed. SERCA pumps can be inhibited by general P-type ATPase inhibitors, such as La^3+^ and orthovanadate, and by the specific inhibitor thapsigargin [178].

SERCA2 isoforms are the main variants present during fertilization. In *Xenopus* oocytes, endogenous SERCA2 is found in cortical clusters [179]. Exogenous expression of avian SERCA1 results in IP3-induced Ca^2+^ oscillations of higher frequency and shorter duration, indicating that SERCA isoforms probably are important in Ca^2+^ regulation at fertilization in frogs [180]. In mouse eggs, SERCA2B is the major isoform expressed [181]. A role for SERCA in sustaining Ca^2+^ oscillations following fertilization was demonstrated by showing that thapsigargin treatment severely reduces the magnitude and duration of the first Ca^2+^ transient and decreases persistence of oscillations [76]. Whereas SERCA2B protein levels remain relatively constant during oocyte maturation, a spatial redistribution of the protein occurs, which mimics ER redistribution from a more diffuse pattern in GV oocytes to cortical clusters in eggs [181]. This localization positions the SERCA pump in close proximity to the IP3 receptor, which probably facilitates refilling ER Ca^2+^ stores after depletion following fertilization.

### 5.6. Plasma membrane Ca^2+^-ATPase pumps and sodium–Ca^2+^ exchangers

The PMCA is a P-type Ca^2+^ pump that transports excess intracellular Ca^2+^ outside of the cell to maintain homeostasis. It has high affinity for Ca^2+^ and low transport capacity, and it exchanges Ca^2+^ for H^+^. Its domain structure is similar to other P-type pumps (one transmembrane and three cytoplasmic domains), but it also contains a calmodulin (CaM)-binding site in its C-terminus that functions as an auto-inhibitory domain. Under resting conditions, this domain folds into the ATP-binding site, inhibiting the pump. When intracellular Ca^2+^ increases, Ca^2+^-bound CaM interacts with the CaM-binding site, releasing the auto-inhibitory conformation of the pump and restoring activity [177,178]. PMCA can also be activated by acidic phospholipids through interaction with two binding sites, one in the transmembrane domain, one in the carboxy terminus of the pump. Therefore, membrane composition can affect both activity and localization of PMCA [178,182,183]. Among acidic phospholipids, PIP2 is the most potent activator of PMCA. Moreover, the interaction between PMCA and PIP2 not only activates PMCA enzymatic activity, but also protects PIP2 from hydrolysis by PLC enzymes, thus modulating Ca^2+^ responses [184]. It remains to be determined if this modulatory role of PMCA is relevant in mouse eggs, where the pool of PIP2 hydrolyzed by PLC*ζ* is located in intracellular vesicles rather than in the plasma membrane.

Mammalian PMCA is encoded by four genes (*Atp2b1–4*) that are alternatively spliced to generate around 30 isoforms. PMCA1 and PMCA4 are ubiquitously expressed and considered the housekeeping PMCAs. PMCA2 is expressed in the nervous system and mammary gland, whereas PMCA3 is expressed in the nervous system [178]. In *Xenopus* oocytes and eggs, the presence and localization of PMCA was demonstrated using an antibody that recognizes all four mammalian PMCA isoforms [179]. This study demonstrated a functional role for PMCA in frog oocytes but not eggs. Inhibition of PMCA in fertilized mouse eggs results in Ca^2+^ oscillations in which the first transient has higher amplitude and longer duration, indicating a role for PMCA in extruding excess Ca^2+^ after fertilization [185]. Similarly, Ca^2+^ oscillations triggered by PLC*ζ* injection can be sustained in the absence of extracellular Ca^2+^ if Ca^2+^ efflux through PMCA is blocked by 5 mM Gd^3+^ [181]. Microarray data from mouse oocytes, eggs and preimplantation embryos indicates that *Atp2b1* is the most abundant isoform, followed by *Atp2b3*, while *Atp2b2* and *Atp2b4* are nearly undetectable [186–188]. However, information about the presence and localization of PMCA at the protein level in mouse eggs and its potential role during fertilization is lacking.

Ca^2+^ efflux can occur through PMCA, but also through the Na^+^–Ca^2+^ exchanger (NCX). This transporter, present in the plasma membrane of most cells, exchanges three Na^+^ for one Ca^2+^ and the directionality of the fluxes depends on the membrane potential and the concentration gradients of these cations [189]. NCX contains 10 transmembrane helices, where the ion transport sites and two Ca^2+^-binding regulatory sites reside. In mammals, NCX is encoded by three genes, *Ncx1–3*, with *Ncx1* and *3* undergoing alternative splicing. NCX1 is ubiquitously expressed, NCX2 is expressed in the nervous system, and NCX3 is expressed in the brain and skeletal muscle [189]. The relative importance of NCX activity to overall Ca^2+^ efflux varies depending on the tissue; it is essential in the heart to extrude Ca^2+^ from myocytes during each cardiac cycle, whereas it does not play a major role in the liver [190].

Removal of extracellular Na^+^ causes the NCX to function in the reverse mode, leading to Ca^2+^ influx. This approach has been used to infer the presence of NCX in hamster, mouse, rat and *Xenopus* oocytes [191–195]. In *Xenopus* oocytes, further characterization demonstrated that both NCX1 and NCX3 are expressed in the plasma membrane and are more abundant in the animal hemisphere [195]. In the mouse, experiments using extracellular Na^+^ depletion, as well as Na^+^ ionophore and Ca^2+^ addition after incubation in Ca^2+^-free medium, demonstrated that NCX is present in oocytes and eggs. However, the changes in Ca^2+^ fluxes observed after manipulating Na^+^ gradients are modest, and oocytes can recover from these perturbations, suggesting that NCX does not play an important role under physiological conditions [193].

Both PMCA and NCX undergo changes during oocyte maturation that suggest a decrease in Ca^2+^ efflux in metaphase II eggs compared with GV oocytes. Mouse oocytes respond to an increase in intracellular Na^+^ or a depletion of extracellular Na^+^ by increasing Ca^2+^ influx, due to the NCX ‘reverse mode’ activity. Mouse eggs, on the other hand, fail to respond to these changes in Na^+^ gradients [193]. The reason for maturation-associated downregulation of NCX activity is unknown. In *Xenopus*, PMCA is endocytosed during oocyte maturation, leading to depletion of this ATPase from the plasma membrane and a reduction in Ca^2+^ efflux [196]. Whether or not internalization of PMCA also takes place in mouse oocytes has not been assessed. A decrease in Ca^2+^ efflux during oocyte maturation is another mechanism to maximize Ca^2+^ responses at fertilization.

### 5.7. Ca^2+^ influx channels

We have long been aware that Ca^2+^ influx occurs at fertilization across numerous species. For example, fertilization of sea urchin and marine worm eggs results in a dramatic increase in Ca^2+^ influx within a few minutes as indicated by radiolabeled Ca^2+^ uptake [197,198]. In fertilized hamster and mouse eggs, early electrical recording studies demonstrated that alterations in extracellular Ca^2+^ concentrations change the frequency of periodic hyperpolarizing responses, suggesting that Ca^2+^ influx modulates the frequency of the intracellular Ca^2+^ release events responsible for hyperpolarization [77,191]. In the complete absence of extracellular Ca^2+^, fertilization-induced Ca^2+^ oscillations rapidly cease due to the depletion of ER Ca^2+^ stores [76,191]. In addition to the refilling of ER stores, Ca^2+^ influx in mouse eggs is required for second polar body emission, suggesting that cortical Ca^2+^ signals downstream of Ca^2+^ influx are essential for complete egg activation [185].

There are numerous Ca^2+^-permeable channels on the plasma membrane that when open allow Ca^2+^ to transit from the extracellular milieu down an approximately 10 000-fold concentration gradient into the cytosol. These channels are activated by a wide variety of signals. The voltage-gated Ca^2+^ channels open in response to changes in membrane potential and are best known for their roles in excitable cells such as neurons and cardiomyocytes. The TRP channels are ubiquitously expressed, and most are involved in transmitting sensory inputs or environmental signals. The TRP channels are permeable to multiple cations in addition to Ca^2+^, with variable selectivity toward different cations, and are functional at resting membrane potentials in non-excitable cells [16]. Ubiquitous store-operated Ca^2+^ channels on the plasma membrane are activated in response to depletion of intracellular Ca^2+^ stores, often following PLC-mediated activation of Ca^2+^ release through the IP3 receptor. There is experimental evidence for a role for each of these three channel classes in supporting Ca^2+^ influx necessary for successful egg activation, though the evidence for SOCE is very limited.

SOCE is well established as a major mechanism in somatic cells whereby ER stores are replenished following depletion [199,200]. This mechanism functions across the animal kingdom, including in insects, birds, amphibians and mammals. The major molecular basis for SOCE is that the ER has two molecular sensors of Ca^2+^ store depletion, STIM1 and STIM2, which are ubiquitously expressed single transmembrane proteins with Ca^2+^-binding EF hand domains in the ER lumen. In response to ER store depletion, Ca^2+^ dissociates from the STIM proteins, which then oligomerize and move toward ER-plasma membrane contact sites. STIM oligomers form large clusters, or punctae, that directly interact with plasma membrane ORAI channels, stimulating Ca^2+^ entry. Because depletion of ER Ca^2+^ stores occurs in the many species that depend on IP3-mediated Ca^2+^ release at fertilization, SOCE appears to be an obvious mechanism to replenish ER stores. However, SOCE is inactivated in mammalian cells during mitosis [201] and in *Xenopus* it is inactive during meiosis [202], suggesting that it is unlikely to have a role at fertilization. Both STIM1 and ORAI1 proteins are expressed in mouse eggs [203,204], but two different studies using chemical SOCE inhibitors concluded that SOCE was not required at fertilization in the mouse [185,205]. Other groups used exogenously expressed, fluorescently tagged STIM and/or ORAI proteins to draw conclusions regarding SOCE function in mouse oocytes [204,206]. Both of these studies concluded that SOCE was disabled or minimally active in metaphase II-arrested eggs, consistent with the inhibitor studies. However, conflicting studies in pig oocytes reported that STIM1 and ORAI1 were necessary for egg activation; these studies used knockdown approaches whereby siRNAs were microinjected into immature oocytes to deplete protein levels [207,208]. Unfortunately, neither of these studies tested the impact of protein knockdown on IP3-sensitive ER Ca^2+^ stores in mature oocytes prior to fertilization. Because low ER Ca^2+^ stores before fertilization would be anticipated to result in inhibition of Ca^2+^ oscillatory behaviour following fertilization, these studies were not conclusive. In an effort to definitively determine whether SOCE was required to replenish ER stores at fertilization, a mouse knockout approach was used to generate eggs lacking both STIM1 and STIM2; *Orai1*-null eggs were also tested [68]. *Stim1/Stim2* double knockout eggs had normal ER Ca^2+^ stores and no alterations in Ca^2+^ influx or Ca^2+^ oscillatory behaviour at fertilization relative to controls. Furthermore, eggs lacking ORAI1, the only ORAI channel expressed in mouse eggs, exhibit normal Ca^2+^ handling and homeostasis at fertilization. These findings clearly indicate that in the mouse, SOCE is not the Ca^2+^ influx mechanism responsible for refilling ER stores following fertilization. It remains possible that in other animals SOCE could be used.

Voltage-gated Ca^2+^ channels (Ca_V_) are also important mediators of calcium influx. They are composed of a pore-forming *α*1 subunit associated with an intracellular *β* subunit, and extracellular *α*2 subunit bound by disulfide linkage to a transmembrane *δ* subunit, and sometimes a transmembrane *γ* subunit [17]. Their differential responses to alterations in membrane potential are largely driven by the *α*1 subunits, which are encoded by ten distinct genes in mammals. The voltage-gated Ca^2+^ channels are divided into three families based on their structure and function. The Ca_V_1 channels are sensitive to large changes in membrane potential and tend to support long-lasting, large-conductance Ca^2+^ currents; hence, they are known as ‘L-type’ channels. The Ca_V_2 channels are largely expressed only in brain regions and will not be discussed further here. The Ca_V_3 channels are sensitive to small changes in membrane potential at negative voltages, are rapidly inactivated, and have transient kinetics leading to their characterization as ‘T-type’ channels. An interesting feature of T-type channels is that they can be open even in resting cells at a window of membrane potential between the activating and inactivating potentials; the resulting inward Ca^2+^ current is called ‘window’ current [209].

There is evidence for both L-type and T-type Ca^2+^ channels in supporting Ca^2+^ influx following fertilization in different species. Many marine animals and amphibians use L-type Ca^2+^ channels for this purpose; their opening is triggered by a large change in membrane potential (known as the ‘fertilization potential’) that occurs in response to either sperm–egg binding or fusion [210]. For example, oocytes of the marine worm *Pseudopotamilla occelata* use L-type channels to generate the global Ca^2+^ wave responsible for resumption of meiosis, but not the initial sperm-induced Ca^2+^ increase [211]. In the marine bivalve *Mytilus edulis*, the initial rise in Ca^2+^ following fertilization is dependent on Ca^2+^ influx through L-type channels, whereas internal Ca^2+^ stores provide later Ca^2+^ signals [75]. Limpet oocytes depend entirely on Ca^2+^ influx through L-type channels to provide the egg activation signal [106,212]. Mammalian eggs do not have a positive shift in membrane potential at fertilization, so would not have a mechanism to open L-type channels [213,214]. However, mouse eggs do have a classical T-type current that can be activated under physiological conditions [215,216]. The channel supporting this current is Ca_V_3.2, which is likely to be active through a window current mechanism [217]. Mouse eggs lacking Ca_V_3.2 have lower levels of Ca^2+^ influx during oocyte maturation and following fertilization. Furthermore, female mice whose eggs lack Ca_V_3.2 have reduced litter sizes, suggesting that this channel is critical for efficient egg activation and development to term [217]. However, Ca_V_3.2 cannot be the only Ca^2+^ influx channel active at fertilization in the mouse because in at least some eggs lacking Ca_V_3.2, Ca^2+^ oscillations can persist long-term following fertilization.

In the past few years, attention has turned to the possible roles of TRP channels in supporting Ca^2+^ influx at fertilization. The TRP superfamily is encoded by a large number of distinct protein-coding genes: 28 in mice, 17 in *C. elegans*, and 13 in *Drosophila* [16]. TRP channels are divided into subfamilies based on sequence and topological homology, but the channels within each subfamily are not always activated by similar mechanisms. The canonical TRPs (TRPC1–7 in mammals) are activated downstream of PLC activation and there is some evidence for their involvement in SOCE in somatic cells [218]. It is unlikely that any of these TRPs function to support Ca^2+^ influx at fertilization, at least in the mouse, because mice globally lacking all seven TRPC channels are fully fertile [219]. TRPV3 is a warm temperature-dependent TRP channel whose activity is potentiated by PLC activation [220], suggesting it could function at fertilization. TRPV3 is expressed and functional in mouse eggs, and when stimulated using chemical activators supports Ca^2+^ influx and egg activation [221]. However, *Trpv3*-null mice are fertile, and their eggs have no alterations in sperm-induced Ca^2+^ oscillatory patterns, indicating that this channel is dispensable, though it could contribute to supporting Ca^2+^ influx at fertilization. TRPM7 is a constitutively active ion channel permeable to divalent cations including magnesium and Ca^2+^, but it can be activated by mechanical signals and inhibited by low millimolar magnesium levels [222,223]. TRPM7 is unusual in that it functions both as a channel and a serine/threonine kinase due to the presence of an intracellular kinase domain at the C-terminus [224]. Functional ion channels with characteristics of TRPM7 are expressed on mouse eggs and can support Ca^2+^ influx. Chemical inhibition of these channels in fertilized one-cell embryos impairs development beyond the two-cell stage [225]. To definitively test whether TRPM7 is required to support Ca^2+^ influx at fertilization, *Trpm7* was conditionally deleted from mouse eggs using the Gdf9-cre transgene [89]. Eggs lacking TRPM7 did not have classical TRPM7 ion currents and in addition, their ability to support both spontaneous and ER store depletion-induced Ca^2+^ influx was lost. Furthermore, they had significantly blunted responses to alterations in extracellular Ca^2+^ and magnesium, and reduced Ca^2+^ oscillation frequency and persistence following fertilization. Although female mice carrying *Trpm7*-null eggs were fertile, their heterozygous offspring had abnormalities in weight variance and growth trajectories. These findings indicate that TRPM7 mediates spontaneous and SOCE-like Ca^2+^ influx, is largely responsible for the impact of extracellular Ca^2+^ and magnesium concentrations on Ca^2+^ influx, and is required to support Ca^2+^ influx following fertilization.

Given the previously established role of Ca_V_3.2 in supporting Ca^2+^ influx at fertilization, a double knockout mouse model was established to generate eggs lacking both Ca_V_3.2 and TRPM7 [89]. These eggs had reduced ER Ca^2+^ stores and minimal Ca^2+^ influx following ER store depletion. During approximately the first hour following fertilization, the double knockout eggs had only one or two Ca^2+^ transients, a finding very similar to the oscillatory pattern observed in wild-type eggs cultured after fertilization in Ca^2+^-free medium. These findings indicate that Ca_V_3.2 and TRPM7 are largely responsible for the Ca^2+^ influx needed to replenish ER stores and to support persistent sperm-induced oscillations. However, many of the double knockout eggs restarted their oscillations again after about an hour, suggesting that a new Ca^2+^ influx mechanism becomes active over time. The restart pattern did not occur when the double knockout eggs were fertilized by ICSI, indicating that the new influx mechanism depends on either sperm–egg plasma membrane interaction or fusion. Female mice carrying Ca_V_3.2/TRPM7 double knockout eggs had reduced litter sizes explained by differences in implantation or post-implantation development and the offspring had increased weight variability relative to controls. These findings indicate that together, Ca_V_3.2 and TRPM7 serve as essential mediators of Ca^2+^ influx following fertilization in mice, though additional channels such as TRPV3 could also contribute. Whether or not they serve a similar function in eggs of other mammals is not known.

**Figure 4.**
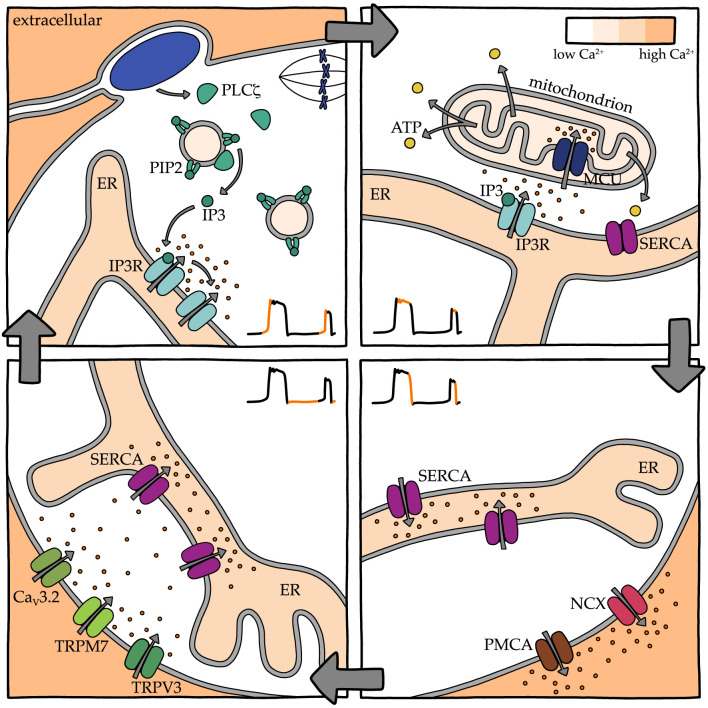
Cycle of Ca^2+^ transient generation in mammalian eggs at fertilization. Starting at the top left, the large grey arrows show temporal order. For each panel, the cytoplasmic Ca^2+^ trace is coloured orange at the portions of the trace that are generated mainly due to the steps illustrated in that panel. Top left: Sperm PLC*ζ* acts on PIP2 in intracellular vesicles to generate IP3, which stimulates IP3R-mediated Ca^2+^ release and subsequent Ca^2+^-induced Ca^2+^ release. Top right: Ca^2+^ stimulates mitochondrial ATP production; ATP is required for SERCA pump activity. Bottom right: Ca^2+^ is pumped back into the ER through SERCA pumps and out of the egg through PMCA pumps and NCX. Bottom left: Ca^2+^ flows into the cytoplasm through TRMP7, Ca_V_3.2 and TRPV3 channels and is then available for SERCA pumps to replenish ER Ca^2+^ stores in preparation for the next Ca^2+^ release event. Orange dots indicate Ca^2+^ at its destination; small grey arrows show the direction of flow. Ca_V_3.2, T-type voltage-dependent Ca^2+^ channel; IP3, inositol trisphosphate; IP3R, IP3 receptor; MCU, mitochondrial uniporter; NCX, sodium/Ca^2+^ exchanger; PIP2, phosphatidylinositol 4,5-bisphosphate; PLC*ζ*, phospholipase C zeta; PMCA, plasma membrane Ca^2+^ ATPase; SERCA, sarco/endoplasmic reticulum Ca^2+^ ATPase pump; TRPM7, transient receptor potential cation channel subfamily M member 7; TRPV3, transient receptor potential cation channel subfamily V member 3.

There is new evidence that TRP channel function in Ca^2+^ influx at fertilization is conserved in insects and worms. As mentioned above, *Drosophila* egg activation is initiated by Ca^2+^ influx that occurs in response to mechanical pressure during ovulation, though propagation of the Ca^2+^ wave depends on IP3-mediated Ca^2+^ release [63,226]. It turns out that the pressure-induced Ca^2+^ influx is mediated by Trpm, the only *Drosophila* orthologue of mammalian TRPM7 [108]. In *C. elegans*, a sperm plasma membrane TRP channel, TRP-3, serves as a conduit for localized Ca^2+^ influx into the egg following sperm–egg fusion [62]. In the absence of sperm TRP-3, eggs still activate following sperm–egg fusion but their global Ca^2+^ wave is delayed and abnormally shaped. Taken together, these findings indicate that TRP channels are essential modulators of Ca^2+^ signals required for the activation of development in both protostomes and deuterostomes. A schematic illustrating the Ca^2+^ release, reuptake and efflux mechanisms active in mouse eggs following fertilization is shown in figure 4.

## 6. Artificial activation

Eggs of numerous species can be activated in the absence of sperm, known as parthenogenetic activation, by artificially increasing cytoplasmic Ca^2+^ levels. Although the importance of Ca^2+^ was not known at the time, Jacques Loeb found during a series of studies beginning in the late 1800's that placement of sea urchin eggs into hypertonic solutions resulted in the elevation of the fertilization envelope, an initial step of fertilization [227]. It was later discovered that many different stimuli could be used to induce ‘artificial parthenogenesis' in marine animals, including electrical currents, various acids and ultraviolet light [228–230]. Based on these studies, Gregory Pincus found that mammalian eggs could undergo parthenogenetic activation when exposed to hypertonic solutions, acid or heat [231]. This body of work paved the way for numerous subsequent experimental studies using parthenogenetic activation to answer basic questions in developmental biology, including the finding that artificially activated mammalian eggs only develop part way due to imprinting [232,233]. More recently, parthenogenetic activation has been used for clinical applications during assisted reproductive technologies (ART) in humans (ICSI failure) and domestic animals [to improve egg activation following ICSI or during cloning (somatic cell nuclear transfer, SCNT)]. The present section aims to review the different stimuli used for egg activation in mammals, with emphasis on the latest methods reported and the role of Ca^2+^ in these artificial processes.

**Figure 5.**
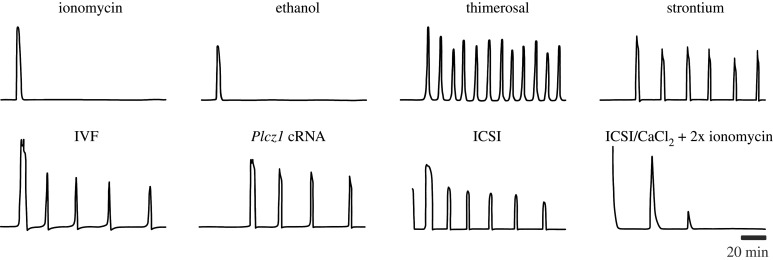
Representative Ca^2+^ traces resulting from egg activation by various methods. Top row, chemical inducers of artificial egg activation (AEA) in bovine eggs [234]. Bottom row, *in vitro* fertilization (IVF) [68], *Plcz1* cRNA injection [123] and intracytoplasmic sperm injection (ICSI) [235] in mouse eggs; ICSI using sperm lacking PLC*ζ* activity, in medium containing CaCl_2_, followed by two treatments with ionomycin in human egg [235]. Scale bar applies to all traces. Traces adapted from indicated references.

Many different methods are used to induce the rise in intracellular Ca^2+^ levels that artificially triggers cell cycle resumption and embryo development. We refer to these methods as ‘artificial egg activation’ (AEA) because they are often used in the presence of the paternal genome and therefore are not the same as parthenogenetic activation. Whereas some of these methods mimic the physiological repetitive Ca^2+^ increases observed in mammals, others simply induce a single large rise in Ca^2+^ that is sufficient to activate the downstream effectors necessary to complete egg activation. Finally, alternative methods completely bypass the Ca^2+^-dependent steps to induce AEA by activating directly the necessary downstream signalling pathways; these methods will not be discussed here as they are outside the scope of this review. Typical Ca^2+^ traces observed in mammalian eggs following the various methods of egg activation are shown in figure 5.

### 6.1. Physical stimuli

Mechanical activation can be achieved efficiently in a small number of species. Under physiological conditions, *Drosophila* egg activation is initiated by mechanosensitive ion channels. These eggs can be artificially activated by osmotic swelling after exposure to hypotonic water or by applying hydrostatic pressure to mature eggs [226]. Ca^2+^ entry provoked after mechanical membrane disruption of frog eggs also results in AEA.

Electrostimulation has been used in several species to trigger egg activation [82,83,236]. This strategy involves the use of high-voltage electrical pulses to induce transitory pores in cellular membranes. The resulting influx of Ca^2+^ from the extracellular space causes a transient rise in intracellular Ca^2+^. The advantage of this method is that the Ca^2+^ rises can be controlled completely based on the equipment settings. Traditionally, electrical pulse length was from milliseconds to microseconds, but these relatively long-duration electric pulses can open large pores that are irreversible and lead to cell death [237]. New technology allows the use of electrical pulses with nanosecond duration. The main advantage to this approach is that nanosecond pulses preferentially affect intracellular membranes with almost no effect on the plasma membrane [238]. In mouse eggs, nanosecond pulsed electric fields (nsPEF) support Ca^2+^ efflux from the ER and even induce spontaneous Ca^2+^ oscillations while maintaining the integrity of the plasma membrane [239]. This procedure results in a high activation rate and a significant improvement in parthenogenetic embryo development. The use of nsPEF for egg activation in species other than mouse has not been tested.

### 6.2. Chemical stimuli

Chemicals are the most popular stimuli used for egg activation across most species. Different responses are registered after egg exposure to chemical agents, varying from a single Ca^2+^ transient to spontaneous Ca^2+^ oscillations. The characteristic responses serve as a reference for their classification (figure 5).

#### 6.2.1. Single peak

Ca^2+^-selective ionophores are the most commonly used chemicals for egg activation. Ionophores are natural lipid-soluble agents that reversibly bind ions. Ca^2+^ ionophores such as ionomycin and calcimycin (A23187) bind Ca^2+^ in a 1 : 1 ratio and facilitate its transport across the cell membrane. In somatic cells, exposure to calcimycin promotes Ca^2+^ efflux from intracellular stores as well as Ca^2+^ influx from the extracellular space [76], whereas ionomycin at low concentrations acts primarily on intracellular membranes but also induces extracellular Ca^2+^ influx [240]. In mouse, human and bovine eggs, treatment with either ionophore leads to a rapid rise in intracellular Ca^2+^ followed by a slow decline over the course of several minutes. Ca^2+^ mobilization stops after drug washout, resulting in a transient effect, though additional ionophore treatments can result in additional Ca^2+^ increases in the same egg. Ca^2+^ ionophores are used in some human ART clinics to artificially activate eggs that fail to activate following ICSI. Although both ionophores function similarly, a direct comparison between ionomycin and commercially available ‘ready to use’ A23187 revealed that ionomycin is more efficient in activating both mouse and human eggs [241]. However, even an ICSI protocol in which the sperm is injected in medium containing CaCl_2_ followed by two treatments with ionomycin does not truly mimic a physiological Ca^2+^ oscillatory pattern (figure 5).

Eggs also can be artificially activated with 7% ethanol, which results in a dramatic rise in intracellular Ca^2+^, mainly due to IP3-mediated Ca^2+^ release from intracellular stores [234,242]. Ethanol activation has been successfully achieved in mouse, bovine and porcine; however, ethanol was not effective for human egg activation [234,242–244]. In bovine, the Ca^2+^ rise induced by ethanol is longer than that observed following ionophore-mediated activation and is associated with a higher activation rate [234]. Ethanol effects cease upon drug washout, comparable to ionophore treatment.

A single increase in the egg intracellular Ca^2+^ level results in relatively low rates of egg activation in mammals. Presumably, a transient rise in Ca^2+^ levels leads to inactivation of maturation promoting factor, a key Ca^2+^ target whose inactivation is essential for resumption of meiosis, but is not sufficient to sustain late events of egg activation that require additional Ca^2+^ signals [82,245,246]. For this reason, ionophore- or ethanol-mediated activation are usually followed by a treatment with protein synthesis or protein kinase inhibitors to improve activation rates [247].

#### 6.2.2. Multiple peaks

In mammals, the information to elicit egg activation is encoded in sperm-induced repetitive Ca^2+^ signals. Therefore, chemicals that trigger Ca^2+^ oscillations activate eggs more efficiently than chemicals that induce a single rise, even though the eggs are flexible regarding the exact Ca^2+^ oscillatory pattern for activation. Strontium is the compound of choice for egg activation in rodents, but its efficacy in other species is controversial. The discovery that strontium activates mouse eggs was made inadvertently during experiments designed to examine the ability of various divalent cations to substitute for Ca^2+^ during sperm capacitation [248]. In the mouse egg, strontium induces prolonged Ca^2+^ oscillations and supports preimplantation embryo development. Strontium-induced activation is mediated by the IP3 receptor, probably through its sensitization to Ca^2+^, and depends on the presence of TRPV3 in the egg [221]. A long first Ca^2+^ transient is observed immediately after eggs are exposed to strontium, which is followed by repetitive smaller spikes resembling the oscillatory pattern observed after IVF [249]. In bovine, pig and horse, strontium can induce egg activation; however, the activation rate and embryo development are lower relative to other activation methods [247,250–252]. In large animals, Ca^2+^ dynamics after strontium-mediated activation remain unexplored, but it is likely that the activation failure is related to inefficient induction of Ca^2+^ oscillations. Strontium does not induce Ca^2+^ oscillations in human eggs and, consequently, fails to trigger egg activation [253,254].

Thimerosal is a mercury-containing organic compound that induces Ca^2+^ release in several cell types. It evokes repetitive Ca^2+^ oscillations in eggs, as was first observed in experiments with golden hamster eggs [255]. Thimerosal increases the sensitivity of the IP3 receptor to Ca^2+^ by oxidation of sulfhydryl groups, resulting in multiple Ca^2+^ oscillations with similar dynamics to sperm-induced Ca^2+^ signalling, but egg activation is not observed with use of thimerosal alone [256]. Thimerosal has a deleterious effect on eggs due to tubulin oxidation, which prevents its polymerization and impairs spindle formation [257]. However, a sequential treatment of thimerosal followed by dithiothreitol (DTT), a reducing reagent, prevents tubulin oxidation and results in a single intracellular Ca^2+^ rise that leads to egg activation in pigs [258]. Surprisingly, although the use of DTT stops thimerosal induced Ca^2+^ signalling in the egg, it enhances Ca^2+^ response after IVF or other activators of Ca^2+^-spiking, which highlights a difference in the mechanism of Ca^2+^ release [256]. In cow and human eggs, thimerosal also evokes Ca^2+^ oscillations, whereas in sea urchin, thimerosal induces a single Ca^2+^ wave that closely resembles fertilization signalling [234,259,260]. The use of thimerosal for egg activation is not widespread, probably due to both safety concerns about mercury and the need for a reducing reagent that results in the premature termination of the Ca^2+^ oscillations.

#### 6.2.3. ‘Physiological’ stimuli

Even though we do not fully understand the long-term effects of AEA, methods that best mimic sperm-induced Ca^2+^ responses are likely to be more effective than those that induce non-physiological patterns. Therefore, microinjection of eggs with sperm extracts, PLC*ζ* or IP3 to trigger egg activation is more attractive for clinical applications. Sperm extracts were first proposed for egg activation after it was shown that they had this activity. Activation rates of cloned horse embryos using stallion sperm extracts were significantly improved relative to other methods [261]. Recently, the use of sperm extracts for activation of SCNT reconstructed embryos in zebrafish was reported to improve the early development of parthenogenetic and cloned embryos; however, it remains uncertain if the Ca^2+^ response is similar to that induced by sperm [262].

To avoid using complex biological extracts, the use of cRNA encoding PLC*ζ* emerged as a suitable solution. Bovine SCNT embryos activated by *PLCZ1* cRNA microinjection exhibited not only a similar Ca^2+^ oscillatory pattern but also a similar gene expression profile when compared with IVF controls [263]. Moreover, ICSI and parthenogenetic activation rates, as well as embryo development, were improved with *PLCZ1* cRNA microinjection in human eggs as compared with ionomycin-mediated activation [264]. Similar results were reported for mouse eggs [265].

There are several concerns regarding the use of *PLCZ1* cRNA and sperm extracts for clinical use. First, it is not possible to accurately control the expression of PLC*ζ* protein from microinjected cRNA [264]. In addition, governmental policies closely regulate the use of biological extracts and nucleic acids for clinical treatments because of the potential for infectious or hereditary long-term outcomes. Both considerations can be bypassed by the use of recombinant PLC*ζ* protein. In this regard, microinjection of human eggs with recombinant human PLC*ζ* protein produced in *E. coli* overcomes clinical ICSI failure and supports embryo development to the blastocyst stage [266]. Despite these promising results, recombinant PLC*ζ* is not yet commercially available.

The increasing use of artificial reproductive technologies has sparked interest among researchers and human as well as animal clinics about AEA. However, special caution needs to be taken due to the concerns regarding possible long-term effects on offspring health of abnormal Ca^2+^ signalling after IVF. Further investigation is required to design accurate methods for egg activation that mimic physiological events, sustain embryonic development and ensure offspring health.

## 7. Post-ovulatory ageing

In mammals, after ovulation there is a defined time window for successful fertilization and development after which deterioration of the egg takes place, known as post-ovulatory ageing. Post-ovulatory aged eggs display increased fragmentation, reduced fertilization and developmental potential and, when successfully fertilized, abnormalities in the offspring [267]. Eggs that undergo post-ovulatory ageing *in vitro* or *in vivo* have altered Ca^2+^ responses triggered by many stimuli including sperm, IP3 and PLC*ζ*. Both the amplitude and the rate of rise of Ca^2+^ transients are lower in post-ovulatory aged eggs [268–271]. The likely reasons for these changes are that the size of ER Ca^2+^ stores is smaller and that the IP3 receptor sensitivity and level of phosphorylation are decreased in aged eggs [271,272]. Also, the spatial organization of the IP3 receptor is altered with ageing in that the cortical IP3R clusters normally present in freshly ovulated MII eggs are dispersed [272]. IP3 receptor sensitivity, phosphorylation and cortical clustering are all associated with efficient Ca^2+^ release [33]. Inhibition of IP3R1 phosphorylation in mouse eggs results in a decrease in both amplitude and persistence of Ca^2+^ oscillations [38]. Consistent with this finding, the total duration of Ca^2+^ oscillations is also shorter in post-ovulatory aged eggs [269,270,273]. Post-ovulatory ageing also results in higher frequency of Ca^2+^ oscillations [269,271,273]. An increase in Ca^2+^ influx triggers more frequent Ca^2+^ oscillations [205]. Interestingly, in bovine eggs, increased Ca^2+^ influx occurs during post-ovulatory ageing; this influx seems to be responsible for the lower developmental potential of aged eggs [274]. Whether Ca^2+^ influx is altered in post-ovulatory aged eggs of other mammalian species is currently unknown. The rate of decrease of Ca^2+^ transients is also smaller in post-ovulatory aged eggs, probably because SERCA2 mRNA and protein levels are reduced [269,271,273]. Mitochondrial function is hampered in post-ovulatory aged eggs, which can affect Ca^2+^ oscillations in at least two ways [272,273]. ATP production, necessary for SERCA and PMCA pump activity, is reduced and Ca^2+^ uptake into the mitochondria is probably diminished. Alterations in these two processes would result in a decline in the eggs' ability to buffer cytosolic Ca^2+^ and could explain why the decrease in Ca^2+^ levels after each transient is slower in aged eggs.

An IP3R1 protein fragment generated by caspase-mediated cleavage may also cause altered Ca^2+^ signalling during post-ovulatory ageing. In Jurkat cells undergoing apoptosis, caspase-3 cleaves IP3R1, generating a 95 kDa C-terminal fragment that contains all transmembrane domains and the channel pore [275]. *In vitro* aged eggs contain this 95 kDa fragment, which is not present in freshly ovulated eggs. Injection of mRNA encoding this fragment into fresh eggs results in fragmentation and impaired Ca^2+^ oscillations, suggesting that processing of the IP3 receptor is responsible, at least in part, for altered Ca^2+^ signalling during ageing [276]. Interestingly, whereas Ca^2+^ oscillations trigger egg activation in freshly ovulated eggs, they cause apoptosis in post-ovulatory aged eggs [277]. The fusion of an *in vitro* aged, activated egg with a freshly ovulated egg decreases fragmentation and improves developmental potential, indicating that some cytoplasmic factors are lost during ageing. One such molecule is the anti-apoptotic factor *Bcl-2*, whose mRNA and protein levels are reduced in aged eggs [270].

The process of post-ovulatory ageing and its detrimental effects on fertilization and embryonic development are particularly relevant for ART. An extended period of time can occur between egg retrieval and insemination, especially in ‘rescue ICSI’ cases, in which eggs that failed to fertilize following insemination are later injected with sperm. Further elucidation of the mechanisms involved in post-ovulatory ageing is key to developing protocols to alleviate this age-dependent deterioration of fertilization and developmental potential during ART procedures.

## 8. Some remaining questions

Throughout the text, we pointed out areas of uncertainty or controversy regarding how Ca^2+^ signals are modulated at fertilization. Here, we will highlight a few new areas that could have significant impacts on these Ca^2+^ signals but have not been studied in depth. First, altered metabolic states have broad-ranging impacts on cellular physiology through changed dynamics of reactive oxygen species. These impacts probably include Ca^2+^ handling, for example, by affecting the amount of Ca^2+^ in intracellular stores, production of ATP necessary for active Ca^2+^ transport or changing downstream intracellular signalling pathways. What are the impacts of altered adult metabolic status on fertilization-induced Ca^2+^ signals, and do these changes impact offspring health? Second, there is voluminous support in somatic cells for the idea that membrane contact sites are regions critical for Ca^2+^ exchange between organelles and/or the extracellular space. Furthermore, lipid-transport proteins at membrane contact sites support the rapid transport of phospholipids, perhaps including PIP2, between the ER and other membrane-bound compartments. Both of these functions suggest a role for membrane contact sites and their associated proteins in regulating Ca^2+^ signals at fertilization, but to date there is no such evidence. Third, to what extent do organelles besides the ER function in regulating Ca^2+^ signals at fertilization? There is good evidence that Ca^2+^ released at fertilization impacts mitochondrial ATP production [278], but are mitochondria and/or vesicles of the endolysosomal system important for Ca^2+^ regulation as well? For mammals, what is the nature of the intracellular vesicles where the PIP2 substrate for IP3 synthesis is found?

Finally, although the wealth of information we have regarding Ca^2+^ signalling in external fertilizers was derived from generally physiological experimental conditions, almost all that we know regarding internal fertilizers, particularly mammals, has been obtained from experiments performed in culture dishes. Two exceptions to this statement are *C. elegans* and *Drosophila* in which genetic tools, combined with the relative transparency of the relevant organs, allowed *in vivo* observations of Ca^2+^ signals at fertilization [62,63]. We have long assumed that findings from *in vitro* studies reflect what occurs *in vivo*, but this assumption was strongly challenged by the recent findings that mouse sperm lacking PLC*ζ* produce offspring despite highly abnormal patterns of Ca^2+^ signalling when studied *in vitro* [91,92]. These findings suggested that either the *in vivo* patterns were different from *in vitro* or that studies defining the necessity for a prolonged series of Ca^2+^ oscillations to support full-term development were only applicable *in vitro*. This question could be resolved with appropriate genetically encoded Ca^2+^ indicators combined with intravital imaging. These and other questions will keep scientists interested in understanding the Ca^2+^ signals responsible for the initiation of development busy for the foreseeable future.

## Data accessibility

This article has no additional data.

## References

[1] Lillie F 1919 Problems of fertilization. Chicago, IL: University of Chicago Press.

[2] Dalcq A 1928 Le rôle du calcium et du potassium dans l'entrée en maturation de l'oeuf de pholade (*Barnea candida*). Protoplasma 4, 18–44. (10.1007/BF01607955)

[3] Ridgway EB, Gilkey JC, Jaffe LF 1977 Free calcium increases explosively in activating medaka eggs. Proc. Natl Acad. Sci. USA 74, 623–627. (10.1073/pnas.74.2.623) 322135 PMC392344

[4] Gilkey JC, Jaffe LF, Ridgway EB, Reynolds GT 1978 A free calcium wave traverses the activating egg of the medaka, *Oryzias latipes*. J. Cell Biol. 76, 448–466. (10.1083/jcb.76.2.448) 10605450 PMC2109987

[5] Shimomura O, Johnson FH, Saiga Y 1962 Extraction, purification and properties of aequorin, a bioluminescent protein from the luminous hydromedusan, *Aequorea*. J. Cell. Comp. Physiol. 59, 223–239. (10.1002/jcp.1030590302) 13911999

[6] Steinhardt R, Zucker R, Schatten G 1977 Intracellular calcium release at fertilization in the sea urchin egg. Dev. Biol. 58, 185–196. (10.1016/0012-1606(77)90084-7) 326602 PMC4351706

[7] Kashir J, Deguchi R, Jones C, Coward K, Stricker SA 2013 Comparative biology of sperm factors and fertilization-induced calcium signals across the animal kingdom. Mol. Reprod. Dev. 80, 787–815. (10.1002/mrd.22222) 23900730

[8] Dresselhaus T, Sprunck S, Wessel GM 2016 Fertilization mechanisms in flowering plants. Curr. Biol. 26, R125–R139. (10.1016/j.cub.2015.12.032) 26859271 PMC4934421

[9] Decuypere JP, Bultynck G, Parys JB 2011 A dual role for Ca^2+^ in autophagy regulation. Cell Calcium 50, 242–250. (10.1016/j.ceca.2011.04.001) 21571367

[10] Ljubojevic S, Bers DM 2015 Nuclear calcium in cardiac myocytes. J. Cardiovasc. Pharmacol. 65, 211–217. (10.1097/FJC.0000000000000174) 25329749 PMC4355307

[11] Di Capite J, Ng SW, Parekh AB. 2009 Decoding of cytoplasmic Ca^2+^ oscillations through the spatial signature drives gene expression. Curr. Biol. 19, 853–858. (10.1016/j.cub.2009.03.063) 19375314

[12] Berridge MJ, Lipp P, Bootman MD 2000 The versatility and universality of calcium signalling. Nat. Rev. Mol. Cell Biol. 1, 11–21. (10.1038/35036035) 11413485

[13] Krebs J, Agellon LB, Michalak M 2015 Ca^2+^ homeostasis and endoplasmic reticulum (ER) stress: an integrated view of calcium signaling. Biochem. Biophys. Res. Commun. 460, 114–121. (10.1016/j.bbrc.2015.02.004) 25998740

[14] Lloyd-Evans E, Waller-Evans H 2019 Lysosomal Ca^2+^ homeostasis and signaling in health and disease. Cold Spring Harb. Perspect. Biol. 12, a035311 (10.1101/cshperspect.a035311) PMC7263086 31653642

[15] Romero-Garcia S, Prado-Garcia H 2019 Mitochondrial calcium: transport and modulation of cellular processes in homeostasis and cancer. Int. J. Oncol. 54, 1155–1167. (10.3892/ijo.2019.4696) 30720054

[16] Venkatachalam K, Montell C 2007 TRP channels. Annu. Rev. Biochem. 76, 387–417. (10.1146/annurev.biochem.75.103004.142819) 17579562 PMC4196875

[17] Catterall W 2000 Structure and regulation of voltage-gated Ca^2+^ channels. Annu. Rev. Cell Dev. Biol. 16, 555 (10.1146/annurev.cellbio.16.1.521) 11031246

[18] Smyth JT, DeHaven WI, Jones BF, Mercer JC, Trebak M, Vazquez G, Putney JW 2006 Emerging perspectives in store-operated Ca^2+^ entry: roles of Orai, Stim and TRP. Biochim. Biophys. 1763, 1147–1160. (10.1016/j.bbamcr.2006.08.050) 17034882

[19] Phillips MJ, Voeltz GK 2016 Structure and function of ER membrane contact sites with other organelles. Nat. Rev. Mol. Cell Biol. 17, 69–82. (10.1038/nrm.2015.8) 26627931 PMC5117888

[20] Soboloff J et al 2007 TRPC channels: integrators of multiple cellular signals. In Transient receptor potential (TRP) channels (eds Flockerzi V, Nilius B), pp. 575–591. Berlin, Germany: Springer. 10.1007/978-3-540-34891-7_34 17217080

[21] Eichmann TO, Lass A 2015 DAG tales: the multiple faces of diacylglycerol—stereochemistry, metabolism, and signaling. Cell. Mol. Life Sci. 72, 3931–3952. (10.1007/s00018-015-1982-3) 26153463 PMC4575688

[22] Morgan AJ 2016 Ca^2+^ dialogue between acidic vesicles and ER. Biochem. Soc. Trans. 44, 546–553. (10.1042/BST20150290) 27068968

[23] Palty R et al 2010 NCLX is an essential component of mitochondrial Na^+^/Ca^2+^ exchange. Proc. Natl Acad. Sci. USA 107, 436–441. (10.1073/pnas.0908099107) 20018762 PMC2806722

[24] Luongo TS et al 2017 The mitochondrial Na^+^/Ca^2+^ exchanger is essential for Ca^2+^ homeostasis and viability. Nature 545, 93–97. (10.1038/nature22082) 28445457 PMC5731245

[25] von Stetina JR, Orr-Weaver TL. 2011 Developmental control of oocyte maturation and egg activation in metazoan models. Cold Spring Harb. Perspect. Biol. 3, 1–19. (10.1101/cshperspect.a005553) PMC3179337 21709181

[26] Chiba K, Kado RT, Jaffe LA 1990 Development of calcium release mechanisms during starfish oocyte maturation. Dev. Biol. 140, 300–306. (10.1016/0012-1606(90)90080-3) 2373255

[27] Fujiwara T, Nakada K, Shirakawa H, Miyazaki S 1993 Development of inositol trisphosphate-induced calcium release mechanism during maturation of hamster oocytes. Dev. Biol. 156, 69–79. (10.1006/dbio.1993.1059) 8383620

[28] Mehlmann LM, Kline D 1994 Regulation of intracellular calcium in the mouse egg: calcium release in response to sperm or inositol trisphosphate is enhanced after meiotic maturation. Biol. Reprod. 51, 1088–1098. (10.1095/biolreprod51.6.1088) 7888488

[29] Jones KT, Carroll J, Whittingham DG 1995 Ionomycin, thapsigargin, ryanodine, and sperm induced Ca^2+^ release increase during meiotic maturation of mouse oocytes. J. Biol. Chem. 270, 6671–6677. (10.1074/jbc.270.12.6671) 7896808

[30] Mehlmann LM, Mikoshiba K, Kline D 1996 Redistribution and increase in cortical inositol 1,4,5-trisphosphate receptors after meiotic maturation of the mouse oocyte. Dev. Biol. 180, 489–498. (10.1006/dbio.1996.0322) 8954721

[31] Wakai T, Vanderheyden V, Yoon SY, Cheon B, Zhang N, Parys JB, Fissore RA 2012 Regulation of inositol 1,4,5-trisphosphate receptor function during mouse oocyte maturation. J. Cell. Physiol. 227, 705–717. (10.1002/jcp.22778) 21465476 PMC3144990

[32] Tombes RM, Simerly C, Borisy GG, Schatten G 1992 Meiosis, egg activation, and nuclear envelope breakdown are differentially reliant on Ca^2+^, whereas germinal vesicle breakdown is Ca^2+^ independent in the mouse oocyte. J. Cell Biol. 117, 799–811. (10.1083/jcb.117.4.799) 1577859 PMC2289470

[33] Wakai T, Fissore RA 2019 Constitutive IP3R1-mediated Ca^2+^ release reduces Ca^2+^ store content and stimulates mitochondrial metabolism in mouse GV oocytes. J. Cell Sci. 132, jcs225441 (10.1242/jcs.225441) 30659110 PMC6382016

[34] Parrington J, Brind S, De Smedt H, Gangeswaran R, Anthony Lai F, Wojcikiewicz R, Carroll J 1998 Expression of inositol 1,4,5-trisphosphate receptors in mouse oocytes and early embryos: the type I isoform is upregulated in oocytes and downregulated after fertilization. Dev. Biol. 203, 451–461. (10.1006/dbio.1998.9071) 9808793

[35] Xu Z, Williams CJ, Kopf GS, Schultz RM 2003 Maturation-associated increase in IP3 receptor type 1: role in conferring increased IP3 sensitivity and Ca^2+^ oscillatory behavior in mouse eggs. Dev. Biol. 254, 163–171. (10.1016/s0012-1606(02)00049-0) 12591238

[36] Fissore RA, Longo FJ, Anderson E, Parys JB, Ducibella T 1999 Differential distribution of inositol trisphosphate receptor isoforms in mouse oocytes. Biol. Reprod. 60, 49–57. (10.1095/biolreprod60.1.49) 9858485

[37] Iwasaki H, Chiba K, Uchiyama T, Yoshikawa F, Suzuki F, Ikeda M, Furuichi T, Mikoshiba K 2002 Molecular characterization of the starfish inositol 1,4,5-trisphosphate receptor and its role during oocyte maturation and fertilization. J. Biol. Chem. 277, 2763–2772. (10.1074/jbc.M108839200) 11687583

[38] Lee B, Vermassen E, Yoon SY, Vanderheyden V, Ito J, Alfandari D, De Smedt H, Parys JB, Fissore RA 2006 Phosphorylation of IP_3_R1 and the regulation of [Ca^2+^]_i_ responses at fertilization: a role for the MAP kinase pathway. Development 133, 4355–4365. (10.1242/dev.02624) 17038520 PMC2909192

[39] Zhang N, Yoon SY, Parys JB, Fissore RA 2015 Effect of M-phase kinase phosphorylations on type 1 inositol 1,4,5-trisphosphate receptor-mediated Ca^2+^ responses in mouse eggs. Cell Calcium 58, 476–488. (10.1016/j.ceca.2015.07.004) 26259730 PMC4631651

[40] Bernhardt ML et al 2015 Regulator of G-protein signaling 2 (RGS2) suppresses premature calcium release in mouse eggs. Development 142, 2633–2640. (10.1242/dev.121707) 26160904 PMC4529029

[41] Jaffe LA, Terasaki M 1994 Structural changes in the endoplasmic reticulum of starfish oocytes during meiotic maturation and fertilization. Dev. Biol. 164, 579–587. (10.1006/dbio.1994.1225) 8045353

[42] Stricker SA, Silva R, Smythe T 1998 Calcium and endoplasmic reticulum dynamics during oocyte maturation and fertilization in the marine worm *Cerebratulus lacteus*. Dev. Biol. 203, 305–322. (10.1006/dbio.1998.9058) 9808782

[43] Kume S, Yamamoto A, Inoue T, Muto A, Okano H, Mikoshiba K 1997 Developmental expression of the inositol 1,4,5-trisphosphate receptor and structural changes in the endoplasmic reticulum during oogenesis and meiotic maturation of *Xenopus laevis*. Dev. Biol. 182, 228–239. (10.1006/dbio.1996.8479) 9070324

[44] Shiraishi K, Okada A, Shirakawa H, Nakanishi S, Mikoshiba K, Miyazaki S 1995 Developmental changes in the distribution of the endoplasmic reticulum and inositol 1,4,5-trisphosphate receptors and the spatial pattern of Ca^2+^ release during maturation of hamster oocytes. Dev. Biol. 170, 594–606. (10.1006/dbio.1995.1239) 7649386

[45] Mann JS, Lowther KM, Mehlmann LM 2010 Reorganization of the endoplasmic reticulum and development of Ca^2+^ release mechanisms during meiotic maturation of human oocytes1. Biol. Reprod. 83, 578–583. (10.1095/biolreprod.110.085985) 20610804 PMC2957155

[46] Mehlmann L, Terasaki M, Jaffe L, Kline D 1995 Reorganization of the endoplasmic reticulum during meiotic maturation of the mouse oocyte. Dev. Biol. 170, 607–615. (10.1006/dbio.1995.1240) 7649387

[47] Ponya Z, Kistof Z, Ciampolini F, Faleri C, Cresti M 2004 Structural change in the endoplasmic reticulum during the *in situ* development and *in vitro* fertilisation of wheat egg cells. Sex Plant Reprod. 17, 177–188. (10.1007/s00497-004-0226-8)

[48] FitzHarris G, Marangos P, Carroll J 2007 Changes in endoplasmic reticulum structure during mouse oocyte maturation are controlled by the cytoskeleton and cytoplasmic dynein. Dev. Biol. 305, 133–144. (10.1016/j.ydbio.2007.02.006) 17368610

[49] Kan R, Yurttas P, Kim B, Jin M, Wo L, Lee B, Gosden R, Coonrod SA 2011 Regulation of mouse oocyte microtubule and organelle dynamics by PADI6 and the cytoplasmic lattices. Dev. Biol. 350, 311–322. (10.1016/j.ydbio.2010.11.033) 21147087 PMC3031771

[50] Kim B, Zhang X, Kan R, Cohen R, Mukai C, Travis AJ, Coonrod SA 2014 The role of MATER in endoplasmic reticulum distribution and calcium homeostasis in mouse oocytes. Dev. Biol. 386, 331–339. (10.1016/j.ydbio.2013.12.025) 24374158 PMC3960596

[51] Boulware MJ, Marchant JS 2005 IP3 receptor activity is differentially regulated in endoplasmic reticulum subdomains during oocyte maturation. Curr. Biol. 15, 765–770. (10.1016/j.cub.2005.02.065) 15854911

[52] Stricker SA 2006 Structural reorganizations of the endoplasmic reticulum during egg maturation and fertilization. Semin. Cell Dev. Biol. 17, 303–313. (10.1016/j.semcdb.2006.02.002) 16546418

[53] Moreno RD, Schatten G, Ramalho-Santos J 2002 Golgi apparatus dynamics during mouse oocyte *in vitro* maturation: effect of the membrane trafficking inhibitor brefeldin A. Biol. Reprod. 66, 1259–1266. (10.1095/biolreprod66.5.1259) 11967185

[54] Payne C, Schatten G 2003 Golgi dynamics during meiosis are distinct from mitosis and are coupled to endoplasmic reticulum dynamics until fertilization. Dev. Biol. 264, 50–63. (10.1016/j.ydbio.2003.08.004) 14623231

[55] Roth MG 1999 Inheriting the Golgi. Cell 99, 559–562. (10.1016/s0092-8674(00)81544-5) 10612391

[56] Wang WA, Agellon LB 2019 Organellar calcium handling in the cellular reticular network. Cold Spring Harb. Perspect. Biol. 11, 1–20. (10.1101/cshperspect.a038265) PMC6886452 31358518

[57] Yu Y, Nomikos M, Theodoridou M, Nounesis G, Lai FA, Swann K 2012 PLC*ζ* causes Ca^2+^ oscillations in mouse eggs by targeting intracellular and not plasma membrane PI(4,5)P 2. Mol. Biol. Cell 23, 371–380. (10.1091/mbc.E11-08-0687) 22114355 PMC3258180

[58] Van Blerkom J 1991 Microtubule mediation of cytoplasmic and nuclear maturation during the early stages of resumed meiosis in cultured mouse oocytes. Proc. Natl Acad. Sci. USA 88, 5031–5035. (10.1073/pnas.88.11.5031) 2052585 PMC51801

[59] Dalton CM, Carroll J 2013 Biased inheritance of mitochondria during asymmetric cell division in the mouse oocyte. J. Cell Sci. 126, 2955–2964. (10.1242/jcs.128744) 23659999 PMC3699109

[60] Yu Y, Dumollard R, Rossbach A, Lai FA, Swann K 2010 Redistribution of mitochondria leads to bursts of ATP production during spontaneous mouse oocyte maturation. J. Cell. Physiol. 224, 672–680. (10.1002/jcp.22171) 20578238 PMC3149123

[61] Stricker SA 1999 Comparative biology of calcium signaling during fertilization and egg activation in animals. Dev. Biol. 211, 157–176. (10.1006/dbio.1999.9340) 10395780

[62] Takayama J, Onami S 2016 The sperm TRP-3 channel mediates the onset of a Ca^2+^ wave in the fertilized *C. elegans* oocyte. Cell Rep. 15, 625–637. (10.1016/j.celrep.2016.03.040) 27068469

[63] Kaneuchi T, Sartain CV, Takeo S, Horner VL, Buehner NA, Aigaki T, Wolfner MF 2015 Calcium waves occur as *Drosophila* oocytes activate. Proc. Natl Acad. Sci. USA 112, 791–796. (10.1073/pnas.1420589112) 25564670 PMC4311822

[64] Tosca L, Glass R, Bronchain O, Philippe L, Ciapa B 2012 PLC*γ*, G-protein of the G*α*q type and cADPr pathway are associated to trigger the fertilization Ca^2+^ signal in the sea urchin egg. Cell Calcium 52, 388–396. (10.1016/j.ceca.2012.06.006) 22784667

[65] Sharma D, Kinsey WH 2008 Regionalized calcium signaling in zebrafish fertilization. Int. J. Dev. Biol. 52, 561–570. (10.1387/ijdb.072523ds) 18649270

[66] Sato KI, Tokmakov AA, Iwasaki T, Fukami Y 2000 Tyrosine kinase-dependent activation of phospholipase C*γ* is required for calcium transient in *Xenopus* egg fertilization. Dev. Biol. 224, 453–469. (10.1006/dbio.2000.9782) 10926780

[67] Harada Y, Kawazoe M, Eto Y, Ueno S, Iwao Y 2011 The Ca^2+^ increase by the sperm factor in physiologically polyspermic newt fertilization: its signaling mechanism in egg cytoplasm and the species-specificity. Dev. Biol. 351, 266–276. (10.1016/j.ydbio.2011.01.003) 21237143

[68] Bernhardt ML, Padilla-Banks E, Stein P, Zhang Y, Williams CJ 2017 Store-operated Ca^2+^ entry is not required for fertilization-induced Ca^2+^ signaling in mouse eggs. Cell Calcium 65, 63–72. (10.1016/j.ceca.2017.02.004) 28222911 PMC5461193

[69] Stephano JL, Gould MC 1997 The intracellular calcium increase at fertilization in *Urechis caupo* oocytes: activation without waves. Dev. Biol. 191, 53–68. (10.1006/dbio.1997.8709) 9356171

[70] Galione A, McDougall A, Busa WB, Willmott N, Gillot I, Whitaker M 1993 Redundant mechanisms of calcium-induced calcium release underlying calcium waves during fertilization of sea urchin eggs. Science 261, 348–352. (10.1126/science.8392748) 8392748

[71] Iwao Y 2012 Egg activation in physiological polyspermy. Reproduction 144, 11–22. (10.1530/REP-12-0104) 22635304

[72] Ueno T, Ohgami T, Harada Y, Ueno S, Iwao Y 2014 Egg activation in physiologically polyspermic newt eggs: involvement of IP3 receptor, PLC*γ*, and microtubules in calcium wave induction. Int. J. Dev. Biol. 58, 315–323. (10.1387/ijdb.130333yi) 25354451

[73] Stricker SA 1996 Repetitive calcium waves induced by fertilization in the nemertean worm *Cerebratulus lacteus*. Dev. Biol. 176, 243–263. (10.1006/dbio.1996.0131) 8660865

[74] Stricker SA, Cline C, Goodrich D 2013 Oocyte maturation and fertilization in marine nemertean worms: using similar sorts of signaling pathways as in mammals, but often with differing results. Biol. Bull. 224, 137–155. (10.1086/BBLv224n3p137) 23995739

[75] Deguchi R, Osanai K, Morisawa M 1996 Extracellular Ca^2+^ entry and Ca^2+^ release from inositol 1,4,5-trisphosphate-sensitive stores function at fertilization in oocytes of the marine bivalve *Mytilus edulis*. Development 122, 3651–3660. 8951080 10.1242/dev.122.11.3651

[76] Kline D, Kline JT 1992 Thapsigargin activates a calcium influx pathway in the unfertilized mouse egg and suppresses repetitive calcium transients in the fertilized egg. J. Biol. Chem. 267, 17 624–17 630. 1387638

[77] Igusa Y, Miyazaki S-I, Yamashita N 1983 Periodic hyperpolarizing responses in hamster and mouse eggs fertilized with mouse sperm. J. Physiol. 340, 633–647. (10.1113/jphysiol.1983.sp014784) 6411906 PMC1199231

[78] Sun FZ, Hoyland J, Huang X, Mason W, Moor RM 1992 A comparison of intracellular changes in porcine eggs after fertilization and electroactivation. Development 115, 947–956. 1451669 10.1242/dev.115.4.947

[79] Nakada K, Shiraishi K, Miyazaki S, Mizuno J, Endo K 1995 Initiation, persistence, and cessation of the series of intracellular Ca^2+^ responses during fertilization of bovine eggs. J. Reprod. Dev. 41, 77–84. (10.1262/jrd.41.77)

[80] Bedford SJ, Kurokawa M, Hinrichs K, Fissore RA 2004 Patterns of intracellular calcium oscillations in horse oocytes fertilized by intracytoplasmic sperm injection: possible explanations for the low success of this assisted reproduction technique in the horse. Biol. Reprod. 70, 936–944. (10.1095/biolreprod.103.021485) 14656727

[81] Swann K, Ozil JP 1994 Dynamics of the calcium signal that triggers mammalian egg activation. Int. Rev. Cytol. 152, 183–222. (10.1016/S0074-7696(08)62557-7) 8206704

[82] Ducibella T, Huneau D, Angelichio E, Xu Z, Schultz RM, Kopf GS, Fissore R, Madoux S, Ozil JP 2002 Egg-to-embryo transition is driven by differential responses to Ca^2+^ oscillation number. Dev. Biol. 250, 280–291. (10.1016/S0012-1606(02)90788-8) 12376103

[83] Ozil JP, Huneau D 2001 Activation of rabbit oocytes: the impact of the Ca^2+^ signal regime on development. Development 128, 917–928. 11222146 10.1242/dev.128.6.917

[84] Ozil JP, Markoulaki S, Toth S, Matson S, Banrezes B, Knott JG, Schultz RM, Huneau D, Ducibella T 2005 Egg activation events are regulated by the duration of a sustained [Ca^2+^]_cyt_ signal in the mouse. Dev. Biol. 282, 39–54. (10.1016/j.ydbio.2005.02.035) 15936328

[85] Tóth S, Huneau D, Banrezes B, Ozil JP 2006 Egg activation is the result of calcium signal summation in the mouse. Reproduction 131, 27–34. (10.1530/rep.1.00764) 16388006

[86] Miao YL, Williams CJ 2012 Calcium signaling in mammalian egg activation and embryo development: the influence of subcellular localization. Mol. Reprod. Dev. 79, 742–756. (10.1002/mrd.22078) 22888043 PMC3502661

[87] Sanders JR, Swann K 2016 Molecular triggers of egg activation at fertilization in mammals. Reproduction 152, R41–R50. (10.1530/REP-16-0123) 27165049

[88] Slusarski DC, Pelegri F 2007 Calcium signaling in vertebrate embryonic patterning and morphogenesis. Dev. Biol. 307, 1–13. (10.1016/j.ydbio.2007.04.043) 17531967 PMC2729314

[89] Bernhardt ML et al 2018 TRPM7 and CaV3.2 channels mediate Ca^2+^ influx required for egg activation at fertilization. Proc. Natl Acad. Sci. USA 115, E10370–E10378. (10.1073/pnas.1810422115) 30322909 PMC6217414

[90] Banrezes B, Sainte-Beuve T, Canon E, Schultz RM, Cancela J, Ozil JP 2011 Adult body weight is programmed by a redox-regulated and energy-dependent process during the pronuclear stage in mouse. PLoS ONE 6, e20388 (10.1371/journal.pone.0029388) 22216268 PMC3247262

[91] Hachem A et al 2017 Plc*ζ* is the physiological trigger of the Ca^2+^ oscillations that induce embryogenesis in mammals but conception can occur in its absence. Development 144, 2914–2924. (10.1242/dev.150227) 28694258 PMC5592814

[92] Nozawa K, Satouh Y, Fujimoto T, Oji A, Ikawa M 2018 Sperm-borne phospholipase C zeta-1 ensures monospermic fertilization in mice. Sci. Rep. 8, 1–10. (10.1038/s41598-018-19497-6) 29358633 PMC5778054

[93] Turner PR, Sheetz MP, Jaffe LA 1984 Fertilization increases the polyphosphoinositide content of sea urchin eggs. Nature 310, 414–415. (10.1038/310414a0) 6087155

[94] Whitaker M, Irvine R. 1984 Inositol 1,4,5-trisphosphate microinjection activates sea urchin eggs. Nature 312, 636–639. (10.1083/jcb.106.2.345)

[95] Busa WB, Nuccitelli R 1985 An elevated free cytosolic Ca^2+^ wave follows fertilization in eggs of the frog, *Xenopus laevis*. J. Cell Biol. 100, 1325–1329. (10.1083/jcb.100.4.1325) 3980584 PMC2113751

[96] Miyazaki S 1988 Inositol 1,4,5-trisphosphate-induced calcium release and guanine nucleotide-binding protein-mediated periodic calcium rises in golden hamster eggs. J. Cell Biol. 106, 345–353. (10.1083/jcb.106.2.345) 3123497 PMC2114965

[97] Kurasawa S, Schultz RM, Kopf GS 1989 Egg-induced modifications of the zona pellucida of mouse eggs: effects of microinjected inositol 1,4,5-trisphosphate. Dev. Biol. 133, 295–304. (10.1016/0012-1606(89)90320-5) 2785065

[98] Thomas TW, Eckberg WR, Dubé F, Galione A 1998 Mechanisms of calcium release and sequestration in eggs of *Chaetopterus pergamentaceus*. Cell Calcium 24, 285–292. (10.1016/S0143-4160(98)90052-5) 9883282

[99] Lee HC, Aarhus R, Walseth TF 1993 Calcium mobilization by dual receptors during fertilization of sea urchin eggs. Science 261, 352–355. (10.1126/science.8392749) 8392749

[100] Chini EN, Beers KW, Dousa TP 1995 Nicotinate adenine dinucleotide phosphate (NAADP) triggers a specific calcium release system in sea urchin eggs. J. Biol. Chem. 270, 3216–3223. (10.1074/jbc.270.7.3216) 7852407

[101] Lim D, Kyozuka K, Gragnaniello G, Carafoli E, Santella L 2001 NAADP+ initiates the Ca^2+^ response during fertilization of starfish oocytes. FASEB J. 15, 2257–2267. (10.1096/fj.01-0157com) 11641253

[102] Moccia F, Lim D, Kyozuka K, Santella L 2004 NAADP triggers the fertilization potential in starfish oocytes. Cell Calcium 36, 515–524. (10.1016/j.ceca.2004.05.004) 15488601

[103] Miyazaki S, Ohmori H, Sasaki S 1975 Action potential and non-linear current–voltage relation in starfish oocytes. J. Physiol. 246, 37–54. (10.1113/jphysiol.1975.sp010879) 1169319 PMC1309403

[104] Shen SS, Buck WR 1993 Sources of calcium in sea urchin eggs during the fertilization response. Dev. Biol. 157, 157–169. (10.1006/dbio.1993.1120) 8482408

[105] Vasilev F, Limatola N, Chun JT, Santella L 2019 Contributions of suboolemmal acidic vesicles and microvilli to the intracellular Ca^2+^ increase in the sea urchin eggs at fertilization. Int. J. Biol. Sci. 15, 757–775. (10.7150/ijbs.28461) 30906208 PMC6429021

[106] Deguchi R 2007 Fertilization causes a single Ca^2+^ increase that fully depends on Ca^2+^ influx in oocytes of limpets (Phylum Mollusca, Class Gastropoda). Dev. Biol. 304, 652–663. (10.1016/j.ydbio.2007.01.017) 17292344

[107] Jaffe LF 1990 The roles of intermembrane calcium in polarizing and activating eggs. In Mechanisms of fertilization: plants to humans (ed. Dale B), pp. 389–417. Berlin, Germany: Springer.

[108] Hu Q, Wolfner MF 2019 The Drosophila Trpm channel mediates calcium influx during egg activation. Proc. Natl Acad. Sci. USA 116, 18 994–19 000. (10.1073/pnas.1906967116) PMC6754564 31427540

[109] Lee KW, Webb SE, Miller AL 1999 A wave of free cytosolic calcium traverses zebrafish eggs on activation. Dev. Biol. 214, 168–180. (10.1006/dbio.1999.9396) 10491266

[110] Lindsay LAL, Hertzler PL, Clark WH 1992 Extracellular Mg^2+^ induces an intracellular Ca^2+^ wave during oocyte activation in the marine shrimp *Sicyonia ingentis*. Dev. Biol. 152, 94–102. (10.1016/0012-1606(92)90159-E) 1628759

[111] Kadamur G, Ross EM 2013 Mammalian phospholipase C. Annu. Rev. Physiol. 75, 127–154. (10.1146/annurev-physiol-030212-183750) 23140367

[112] Carroll DJ, Ramarao CS, Mehlmann LM, Roche S, Terasaki M, Jaffe LA 1997 Calcium release at fertilization in starfish eggs is mediated by phospholipase C*γ*. J. Cell Biol. 138, 1303–1311. (10.1083/jcb.138.6.1303) 9298985 PMC2132564

[113] Runft LL, Jaffe LA 2000 Sperm extract injection into ascidian eggs signals Ca^2+^ release by the same pathway as fertilization. Development 127, 3227–3236. 10887079 10.1242/dev.127.15.3227

[114] Giusti AF, O'Neill FJ, Yamasu K, Foltz KR, Jaffe LA 2003 Function of a sea urchin egg Src family kinase in initiating Ca^2+^ release at fertilization. Dev. Biol. 256, 367–378. (10.1016/S0012-1606(03)00043-5) 12679109

[115] Mehlmann LM, Carpenter G, Rhee SG, Jaffe LA 1998 SH2 domain-mediated activation of phospholipase Cγ is not required to initiate Ca^2+^ release at fertilization of mouse eggs. Dev. Biol. 203, 221–232. (10.1006/dbio.1998.9051) 9806786

[116] Dale B, DeFelice L, Ehrenstein G 1985 Injection of a soluble sperm fraction into sea-urchin eggs triggers the cortical reaction. Experientia 41, 1068–1070. (10.1007/BF01952148) 4018233

[117] Dale B 1988 Primary and secondary messengers in the activation of ascidian eggs. Exp. Cell Res. 177, 205–211. (10.1080/03078698.1994.9674073) 3134248

[118] Swann K 1990 A cytosolic sperm factor stimulates repetitive calcium increases and mimics fertilization in hamster eggs. Development 110, 1295–1302. 2100264 10.1242/dev.110.4.1295

[119] Stice SL, Robl JM 1990 Activation of mammalian oocytes by a factor obtained from rabbit sperm. Mol. Reprod. Dev. 25, 272–280. (10.1002/mrd.1080250309) 2331376

[120] Longo FJ, Lynn JW, McCulloh DH, Chambers EL 1986 Correlative ultrastructural and electrophysiological studies of sperm–egg interactions of the sea urchin, *Lytechinus variegatus*. Dev. Biol. 118, 155–166. (10.1016/0012-1606(86)90083-7) 3770296

[121] McCulloh DH, Chambers EL 1992 Fusion of membranes during fertilization: Increases of the sea urchin egg's membrane capacitance and membrane conductance at the site of contact with the sperm. J. Gen. Physiol. 99, 137–175. (10.1085/jgp.99.2.137) 1613481 PMC2216609

[122] Lawrence Y, Whitaker M, Swann K 1997 Sperm–egg fusion is the prelude to the initial Ca^2+^ increase at fertilization in the mouse. Development 124, 233–241. 9006083 10.1242/dev.124.1.233

[123] Saunders CM, Larman MG, Parrington J, Cox LJ, Royse J, Blayney LM, Swann K, Lai FA 2002 PLC*ζ*: a sperm-specific trigger of Ca^2+^ oscillations in eggs and embryo development. Development 129, 3533–3544. 12117804 10.1242/dev.129.15.3533

[124] Knott JG, Kurokawa M, Fissore RA, Schultz RM, Williams CJ 2005 Transgenic RNA interference reveals role for mouse sperm phospholipase C*ζ* in triggering Ca^2+^ oscillations during fertilization1. Biol. Reprod. 72, 992–996. (10.1095/biolreprod.104.036244) 15601914

[125] Yoon SY et al 2008 Human sperm devoid of PLC, zeta 1 fail to induce Ca^2+^ release and are unable to initiate the first step of embryo development. J. Clin. Invest. 118, 3671–3681. (10.1172/JCI36942.the) 18924610 PMC2567839

[126] Heytens E et al 2009 Reduced amounts and abnormal forms of phospholipase C zeta (PLC*ζ*) in spermatozoa from infertile men. Hum. Reprod. 24, 2417–2428. (10.1093/humrep/dep207) 19584136

[127] Kashir J et al 2012 A maternally inherited autosomal point mutation in human phospholipase C zeta (PLC*ζ*) leads to male infertility. Hum. Reprod. 27, 222–231. (10.1093/humrep/der384) 22095789 PMC3241606

[128] Nomikos M, Elgmati K, Theodoridou M, Calver BL, Cumbes B, Nounesis G, Swann K, Lai FA 2011 Male infertility-linked point mutation disrupts the Ca^2+^ oscillation-inducing and PIP2 hydrolysis activity of sperm PLC*ζ*. Biochem. J. 434, 211–217. (10.1042/BJ20101772) 21204786 PMC3195387

[129] Escoffier J et al 2016 Homozygous mutation of PLCZ1 leads to defective human oocyte activation and infertility that is not rescued by the WW-binding protein PAWP. Hum. Mol. Genet. 25, 878–891. (10.1093/hmg/ddv617) 26721930 PMC4754041

[130] Ferrer-Vaquer A, Barragan M, Freour T, Vernaeve V, Vassena R 2016 PLC*ζ* sequence, protein levels, and distribution in human sperm do not correlate with semen characteristics and fertilization rates after ICSI. J. Assist. Reprod. Genet. 33, 747–756. (10.1007/s10815-016-0718-0) 27138933 PMC4889489

[131] Torra-Massana M, Cornet-Bartolomé D, Barragán M, Durban M, Ferrer-Vaquer A, Zambelli F, Rodriguez A, Oliva R, Vassena R 2019 Novel phospholipase C zeta 1 mutations associated with fertilization failures after ICSI. Hum. Reprod. 34, 1494–1504. (10.1093/humrep/dez094) 31347677

[132] Dai J et al 2020 Novel homozygous variations in PLCZ1 lead to poor or failed fertilization characterized by abnormal localization patterns of PLC*ζ* in sperm. Clin. Genet. 97, 347–351. (10.1111/cge.13636) 31463947

[133] Halet G, Tunwell R, Balla T, Swann K, Carroll J 2002 The dynamics of plasma membrane Ptdlns(4,5)P2 at fertilization of mouse eggs. J. Cell Sci. 115, 2139–2149. 11973355 10.1242/jcs.115.10.2139

[134] Sanders JR, Ashley B, Moon A, Woolley TE, Swann K 2018 PLCζ induced Ca^2+^ oscillations in mouse eggs involve a positive feedback cycle of Ca^2+^ induced InsP_3_ formation from cytoplasmic PIP_2_. Front. Cell Dev. Biol. 6, 36 (10.3389/fcell.2018.00036) 29666796 PMC5891639

[135] Nomikos M, Elgmati K, Theodoridou M, Calver BL, Nounesis G, Swann K, Lai FA 2011 Phospholipase C*ζ* binding to PtdIns(4,5)P 2 requires the XY-linker region. J. Cell Sci. 124, 2582–2590. (10.1242/jcs.083485) 21730019 PMC3138701

[136] Nomikos M et al 2015 Essential role of the EF-hand domain in targeting sperm phospholipase C*ζ* to membrane phosphatidylinositol 4,5-bisphosphate (PIP2). J. Biol. Chem. 290, 29 519–29 530. (10.1074/jbc.M115.658443) PMC4705952 26429913

[137] Nomikos M et al 2017 Male infertility-linked point mutation reveals a vital binding role for the C2 domain of sperm PLC*ζ*. Biochem. J. 474, 1003–1016. (10.1042/BCJ20161057) 28270562

[138] Nomikos M, Elgmati K, Theodoridou M, Georgilis A, Gonzalez-Garcia JR, Nounesis G, Swann K, Lai FA 2011 Novel regulation of PLC*ζ* activity via its XY-linker. Biochem. J. 438, 427–432. (10.1042/BJ20110953) 21767260 PMC3195331

[139] Kouchi Z, Fukami K, Shikano T, Oda S, Nakamura Y, Takenawa T, Miyazaki S 2004 Recombinant phospholipase C*ζ* has high Ca^2+^ sensitivity and induces Ca^2+^ oscillations in mouse eggs. J. Biol. Chem. 279, 10 408–10 412. (10.1074/jbc.M313801200) 14701816

[140] Williams CJ, Schultz RM, Kopf GS 1992 Role of G proteins in mouse egg activation: stimulatory effects of acetylcholine on the ZP2 to ZP2f conversion and pronuclear formation in eggs expressing a functional m1 muscarinic receptor. Dev. Biol. 151, 288–296. (10.1016/0012-1606(92)90233-7) 1577193

[141] Moore G, Kopf G, Schultz R 1993 Complete mouse egg activation in the absence of sperm by stimulation of an exogenous G protein-coupled receptor. Dev. Biol. 159, 669–678. (10.1006/dbio.1993.1273) 8405688

[142] Williams CJ, Mehlmann LM, Jaffe LA, Kopf GS, Schultz RM 1998 Evidence that G(q) family G proteins do not function in mouse egg activation at fertilization. Dev. Biol. 198, 116–127. (10.1006/dbio.1998.8892) 9640335

[143] Igarashi H, Knott JG, Schultz RM, Williams CJ 2007 Alterations of PLCβ1 in mouse eggs change calcium oscillatory behavior following fertilization. Dev. Biol. 312, 321–330. (10.1016/j.ydbio.2007.09.028) 17961538 PMC2170533

[144] Matsu-ura T, Shirakawa H, Suzuki KGN, Miyamoto A, Sugiura K, Michikawa T, Kusumi A, Mikoshiba K 2019 Dual-FRET imaging of IP3 and Ca^2+^ revealed Ca^2+^ -induced IP3 production maintains long lasting Ca^2+^ oscillations in fertilized mouse eggs. Sci. Rep. 9, 1–11. (10.1038/s41598-019-40931-w) 30886280 PMC6423007

[145] Paknejad N, Hite RK 2018 Structural basis for the regulation of inositol trisphosphate receptors by Ca^2+^ and IP3. Nat. Struct. Mol. Biol. 25, 660–668. (10.1038/s41594-018-0089-6) 30013099 PMC6082148

[146] Finch EA, Turner TJ, Goldin SM 1991 Calcium as a coagonist of inositol 1,4,5-trisphosphate-induced calcium release. Science 252, 443–446. (10.1126/science.2017683) 2017683

[147] Marchant JS, Taylor CW 1997 Cooperative activation of IP3 receptors by sequential binding of IP3 and Ca^2+^ safeguards against spontaneous activity. Curr. Biol. 7, 510–518. (10.1016/S0960-9822(06)00222-3) 9210378

[148] Alzayady KJ, Wang L, Chandrasekhar R, Wagner LE, Van Petegem F, Yule DI 2016 Defining the stoichiometry of inositol 1,4,5-trisphosphate binding required to initiate Ca^2+^ release. Sci. Signal. 9, 1–13. (10.1126/scisignal.aad6281) PMC4850551 27048566

[149] Dupont G, Dumollard R 2004 Simulation of calcium waves in ascidian eggs: insights into the origin of the pacemaker sites and the possible nature of the sperm factor. J. Cell Sci. 117, 4313–4323. (10.1242/jcs.01278) 15292399

[150] Berridge MJ 2016 The inositol trisphosphate/calcium signaling pathway in health and disease. Physiol. Rev. 96, 1261–1296. (10.1152/physrev.00006.2016) 27512009

[151] Prole DL, Taylor CW 2019 Structure and function of IP3 receptors. Cold Spring Harb. Perspect. Biol. 11, a035063 (10.1101/cshperspect.a035063) 30745293 PMC6442203

[152] Supattapone S, Danoff SK, Theibert A, Joseph SK, Steiner J, Snyder SH 1988 Cyclic AMP-dependent phosphorylation of a brain inositol trisphosphate receptor decreases its release of calcium. Proc. Natl Acad. Sci. USA 85, 8747–8750. (10.1073/pnas.85.22.8747) 2847175 PMC282538

[153] Ferris CD, Huganir RL, Bredt DS, Cameron AM, Snyder SH 1991 Inositol trisphosphate receptor: phosphorylation by protein kinase C and calcium calmodulin-dependent protein kinases in reconstituted lipid vesicles. Proc. Natl Acad. Sci. USA 88, 2232–2235. (10.1073/pnas.88.6.2232) 1848697 PMC51204

[154] Vanderheyden V, Devogelaere B, Missiaen L, De Smedt H, Bultynck G, Parys JB 2009 Regulation of inositol 1,4,5-trisphosphate-induced Ca^2+^ release by reversible phosphorylation and dephosphorylation. Biochim. Biophys. Acta 1793, 959–970. (10.1038/jid.2014.371) 19133301 PMC2693466

[155] Miyazaki S, Yuzaki M, Nakada K, Shirakawa H, Nakanishi S, Nakade S, Mikoshiba K 1992 Block of Ca^2+^ wave and Ca^2+^ oscillation by antibody to the inositol 1,4,5-trisphosphate receptor in fertilized hamster eggs. Science 257, 251–255. (10.1126/science.1321497) 1321497

[156] Xu Z, Kopf GS, Schultz RM 1994 Involvement of inositol 1,4,5-trisphosphate-mediated Ca^2+^ release in early and late events of mouse egg activation. Development 120, 1851–1859. 7924992 10.1242/dev.120.7.1851

[157] Albrieux M, Sardet C, Villaz M 1997 The two intracellular Ca^2+^ release channels, ryanodine receptor and inositol 1,4,5-trisphosphate receptor, play different roles during fertilization in ascidians. Dev. Biol. 189, 174–185. (10.1006/dbio.1997.8674) 9299112

[158] Yue C, White KL, Reed WA, Bunch TD 1995 The existence of inositol 1,4,5-trisphosphate and ryanodine receptors in mature bovine oocytes. Development 121, 2645–2654. 7545575 10.1242/dev.121.8.2645

[159] Ayabe T, Kopf GS, Schultz RM 1995 Regulation of mouse egg activation: presence of ryanodine receptors and effects of microinjected ryanodine and cyclic ADP ribose on uninseminated and inseminated eggs. Development 121, 2233–2244. 7635066 10.1242/dev.121.7.2233

[160] Macháty Z, Funahashi H, Day BN, Prather RS 1997 Developmental changes in the intracellular Ca^2+^ release mechanisms in porcine oocytes. Biol. Reprod. 56, 921–930. (10.1095/biolreprod56.4.921) 9096874

[161] Sousa M, Barros A, Tesarik J 1996 The role of ryanodine-sensitive Ca^2+^ stores in the Ca^2+^ oscillation machine of human oocytes. Mol. Hum. Reprod. 2, 265–272. (10.1093/molehr/2.4.265) 9238690

[162] Patel S, Joseph SK, Thomas AP 1999 Molecular properties of inositol 1,4,5-trisphosphate receptors. Cell Calcium 25, 247–264. (10.1054/ceca.1999.0021) 10378086

[163] Kume S, Muto A, Aruga J, Nakagawa T, Michikawa T, Furuichi T, Nakade S, Okano H, Mikoshiba K 1993 The *Xenopus* IP3 receptor: structure, function, and localization in oocytes and eggs. Cell 73, 555–570. (10.1016/0092-8674(93)90142-D) 8387895

[164] Baylis HA, Furuichi T, Yoshikawa F, Mikoshiba K, Sattelle DB 1999 Inositol 1,4,5-trisphosphate receptors are strongly expressed in the nervous system, pharynx, intestine, gonad and excretory cell of *Caenorhabditis elegans* and are encoded by a single gene (itr-1). J. Mol. Biol. 294, 467–476. (10.1006/jmbi.1999.3229) 10610772

[165] Taylor CW, Genazzani AA, Morris SA 1999 Expression of inositol trisphosphate receptors. Cell Calcium 26, 237–251. (10.1054/ceca.1999.0090) 10668562

[166] Monkawa T, Miyawaki A, Sugiyama T, Yoneshima H, Yamamoto-Hino M, Furuichi T, Saruta T, Hasegawa M, Mikoshiba K 1995 Heterotetrameric complex formation of inositol 1,4,5-trisphosphate receptor subunits. J. Biol. Chem. 270, 14 700–14 704. (10.1074/jbc.270.24.14700) 7782334

[167] Südhof TC, Newton CL, Archer BT, Ushkaryov YA, Mignery GA 1991 Structure of a novel InsP3 receptor. EMBO J. 10, 3199–3206. (10.1002/j.1460-2075.1991.tb04882.x) 1655411 PMC453043

[168] Taylor CW 2017 Regulation of IP3 receptors by cyclic AMP. Cell Calcium 63, 48–52. (10.1016/j.ceca.2016.10.005) 27836216 PMC5471599

[169] Soulsby MD, Wojcikiewicz RJH 2007 Calcium mobilization via type III inositol 1,4,5-trisphosphate receptors is not altered by PKA-mediated phosphorylation of serines 916, 934, and 1832. Cell Calcium 42, 261–270. (10.1016/j.ceca.2006.12.002) 17257671 PMC1975771

[170] He CL, Damiani P, Ducibella T, Takahashi M, Tanzawa K, Parys JB, Fissore RA 1999 Isoforms of the inositol 1,4,5-trisphosphate receptor are expressed in bovine oocytes and ovaries: the type-1 isoform is down-regulated by fertilization and by injection of adenophostin A. Biol. Reprod. 61, 935–943. (10.1095/biolreprod61.4.935) 10491627

[171] Galione A 2019 NAADP receptors. Cold Spring Harb. Perspect. Biol. 11, a035071 (10.1101/cshperspect.a035071) 31182546 PMC6824237

[172] Churchill GC, Okada Y, Thomas JM, Genazzani AA, Patel S, Galione A 2002 NAADP mobilizes Ca^2+^ from reserve granules, lysosome-related organelles, in sea urchin eggs. Cell 111, 703–708. (10.1016/S0092-8674(02)01082-6) 12464181

[173] Walseth TF, Lin-Moshier Y, Jain P, Ruas M, Parrington J, Galione A, Marchant JS, Slama JT 2012 Photoaffinity labeling of high affinity nicotinic acid adenine dinucleotide phosphate (NAADP)-binding proteins in sea urchin egg. J. Biol. Chem. 287, 2308–2315. (10.1074/jbc.M111.306563) 22117077 PMC3268392

[174] Churchill GC, Galione A 2001 NAADP induces Ca^2+^ oscillations via a two-pool mechanism by priming IP3- and cADPR-sensitive Ca^2+^ stores. EMBO J. 20, 2666–2671. (10.1093/emboj/20.11.2666) 11387201 PMC125473

[175] Morgan AJ, Davis LC, Wagner SKTY, Lewis AM, Parrington J, Churchill GC, Galione A 2013 Bidirectional Ca^2+^ signaling occurs between the endoplasmic reticulum and acidic organelles. J. Cell Biol. 200, 789–805. (10.1083/jcb.201204078) 23479744 PMC3601362

[176] Brailoiu E, Rahman T, Churamani D, Prole DL, Brailoiu GC, Hooper R, Taylor CW, Patel S 2010 An NAADP-gated two-pore channel targeted to the plasma membrane uncouples triggering from amplifying Ca^2+^ signals. J. Biol. Chem. 285, 38 511–38 516. (10.1074/jbc.M110.162073) PMC2992283 20880839

[177] Brini M, Carafoli E 2009 Calcium pumps in health and disease. Physiol. Rev. 89, 1341–1378. (10.1152/physrev.00032.2008) 19789383

[178] Chen J, Sitsel A, Benoy V, Sepúlveda MR, Vangheluwe P 2020 Primary active Ca^2+^ transport systems in health and disease. Cold Spring Harb. Perspect. Biol. 12, a035113 (10.1101/cshperspect.a035113) 31501194 PMC6996454

[179] El-Jouni W, Jang B, Haun S, Machaca K 2005 Calcium signaling differentiation during *Xenopus* oocyte maturation. Dev. Biol. 288, 514–525. (10.1016/j.ydbio.2005.10.034) 16330019

[180] Camacho P, Lechleiter J 1993 Increased frequency of calcium waves in *Xenopus laevis* oocytes that express a calcium-ATPase. Science 260, 226–229. (10.1126/science.8385800) 8385800

[181] Wakai T, Zhang N, Vangheluwe P, Fissore RA 2013 Regulation of endoplasmic reticulum Ca^2+^ oscillations in mammalian eggs. J. Cell Sci. 126, 5714–5724. (10.1242/jcs.136549) 24101727 PMC3860313

[182] Choquette D, Hakim G, Filoteo AG, Plishker GA, Bostwick JR, Penniston JT 1984 Regulation of plasma membrane Ca^2+^ ATPases by lipids of the phosphatidylinositol cycle. Biochem. Biophys. Res. Commun. 125, 908–915. (10.1016/0006-291X(84)91369-X) 6097254

[183] Missiaen L, Raeymaekers L, Wuytack F, Vrolix M, De Smedt H, Casteels R. 1989 Phospholipid-protein interactions of the plasma-membrane Ca^2+^-transporting ATPase. Biochem. J. 263, 687–694. (10.1042/bj2630687) 2532005 PMC1133487

[184] Penniston JT, Padányi R, Pászty K, Varga K, Enyedi L, Enyedi A 2014 Apart from its known function, the plasma membrane Ca^2+^ atpase can regulate Ca^2+^ signaling by controlling phosphatidylinositol 4,5-bisphosphate levels. J. Cell Sci. 127, 72–84. (10.1242/jcs.132548) 24198396

[185] Miao Y-L, Stein P, Jefferson WN, Padilla-Banks E, Williams CJ 2012 Calcium influx-mediated signaling is required for complete mouse egg activation. Proc. Natl Acad. Sci. USA 109, 4169–5174. (10.1073/pnas.1112333109) 22371584 PMC3306664

[186] Zeng F, Baldwin DA, Schultz RM 2004 Transcript profiling during preimplantation mouse development. Dev. Biol. 272, 483–496. (10.1016/j.ydbio.2004.05.018) 15282163

[187] Pan H, O'Brien MJ, Wigglesworth K, Eppig JJ, Schultz RM 2005 Transcript profiling during mouse oocyte development and the effect of gonadotropin priming and development *in vitro*. Dev. Biol. 286, 493–506. (10.1016/j.ydbio.2005.08.023) 16168984

[188] Pan H, Ma P, Zhu W, Schultz RM 2008 Age-associated increase in aneuploidy and changes in gene expression in mouse eggs. Dev. Biol. 316, 397–407. (10.1016/j.ydbio.2008.01.048) 18342300 PMC2374949

[189] Verkhratsky A, Trebak M, Perocchi F, Khananshvili D, Sekler I 2018 Crosslink between calcium and sodium signalling. Exp. Physiol. 103, 157–169. (10.1113/EP086534) 29210126 PMC6813793

[190] Philipson KD, Nicoll DA 2000 Sodium-calcium exchange: a molecular perspective. Annu. Rev. Physiol. 62, 111–133. (10.1146/annurev.physiol.62.1.111) 10845086

[191] Igusa Y, Miyazaki S 1983 Effects of altered extracellular and intracellular calcium concentration on hyperpolarizing responses of the hamster egg. J. Physiol. 340, 611–632. (10.1113/jphysiol.1983.sp014783) 6887062 PMC1199230

[192] Pepperell JR, Kommineni K, Buradagunta S, Smith PJS, Keefe DL 1999 Transmembrane regulation of intracellular calcium by a plasma membrane sodium/calcium exchanger in mouse ova. Biol. Reprod. 60, 1137–1143. (10.1095/biolreprod60.5.1137) 10208975

[193] Carroll J 2000 Na^+^-Ca^2+^ exchange in mouse oocytes: modifications in the regulation of intracellular free Ca^2+^ during oocyte maturation. J. Reprod. Fertil. 118, 337–342. (10.1530/reprod/118.2.337) 10864798

[194] Cui W, Zhang J, Zhang C-X, Jiao G-Z, Zhang M, Wang T-Y, Luo M-J, Tan J-H 2013 Control of spontaneous activation of rat oocytes by regulating plasma membrane Na^+^/Ca^2+^ exchanger activities. Biol. Reprod. 88, 160 (10.1095/biolreprod.113.108266) 23677981

[195] Solís-Garrido LM, Pintado AJ, Andrés-Mateos E, Figueroa M, Matute C, Montiel C 2004 Cross-talk between native plasmalemmal Na^+^/Ca^2+^ exchanger and inositol 1,4,5-trisphosphate-sensitive Ca^2+^ internal store in *Xenopus* oocytes. J. Biol. Chem. 279, 52 414–52 424. (10.1074/jbc.M408872200) 15375168

[196] El-Jouni W, Haun S, Machaca K 2008 Internalization of plasma membrane Ca^2+^-ATPase during *Xenopus* oocyte maturation. Dev. Biol. 324, 99–107. (10.1016/j.ydbio.2008.09.007) 18823969 PMC2632722

[197] Nakazawa T, Asami K, Shoger R, Fujiwara A, Yasumasu I 1970 Ca^2+^ uptake, H^+^ ejection and respiration in sea urchin eggs on fertilization. Exp. Cell Res. 63, 143–146. (10.1016/0014-4827(70)90342-3) 5531477

[198] Johnston RN, Paul M 1977 Calcium influx following fertilization of *Urechis caupo* eggs. Dev. Biol. 57, 364–374. (10.1016/0012-1606(77)90221-4) 17560

[199] Parekh AB, Putney JW 2005 Store-operated calcium channels. Physiol. Rev. 85, 757–810. (10.1152/physrev.00057.2003) 15788710

[200] Feske S, Wulff H, Skolnik EY 2015 Ion channels in innate and adaptive immunity. Annu. Rev. Immunol. 33, 291–353. (10.1146/annurev-immunol-032414-112212) 25861976 PMC4822408

[201] Smyth JT, Putney JW 2012 Regulation of store-operated calcium entry during cell division. Biochem. Soc. Trans. 40, 119–123. (10.1042/BST20110612) 22260676 PMC3332531

[202] Yu F, Sun L, Machaca K 2009 Orai1 internalization and STIM1 clustering inhibition modulate SOCE inactivation during meiosis. Proc. Natl Acad. Sci. USA 106, 17 401–17 406. (10.1073/pnas.0904651106) PMC2765092 19805124

[203] Gómez-Fernández C, Pozo-Guisado E, Gañán-Parra M, Perianes MJ, Alvarez IS, Martín-Romero FJ 2009 Relocalization of STIM1 in mouse oocytes at fertilization: early involvement of store-operated calcium entry. Reproduction 138, 211–221. (10.1530/REP-09-0126) 19470709

[204] Cheon B, Lee HC, Wakai T, Fissore RA 2013 Ca^2+^ influx and the store-operated Ca^2+^ entry pathway undergo regulation during mouse oocyte maturation. Mol. Biol. Cell 24, 1396–1410. (10.1091/mbc.E13-01-0065) 23468522 PMC3639051

[205] Takahashi T, Kikuchi T, Kidokoro Y, Shirakawa H 2013 Ca^2+^ influx-dependent refilling of intracellular Ca^2+^ stores determines the frequency of Ca_2+_ oscillations in fertilized mouse eggs. Biochem. Biophys. Res. Commun. 430, 60–65. (10.1016/j.bbrc.2012.11.024) 23159627

[206] Lee B, Palermo G, Machaca K 2013 Downregulation of store-operated Ca^2+^ entry during mammalian meiosis is required for the egg-to-embryo transition. J. Cell Sci. 126, 1672–1681. (10.1242/jcs.121335) 23424198

[207] Lee K, Wang C, Machaty Z 2012 STIM1 is required for Ca^2+^ signaling during mammalian fertilization. Dev. Biol. 367, 154–162. (10.1016/j.ydbio.2012.04.028) 22565091

[208] Wang C, Lee K, Gajdócsi E, Papp ÁB, Machaty Z 2012 Orai1 mediates store-operated Ca^2+^ entry during fertilization in mammalian oocytes. Dev. Biol. 365, 414–423. (10.1016/j.ydbio.2012.03.007) 22445508

[209] Capiod T 2011 Cell proliferation, calcium influx and calcium channels. Biochimie 93, 2075–2079. (10.1016/j.biochi.2011.07.015) 21802482

[210] Hagiwara S, Jaffe LA 1979 Electrical properties of egg cell membranes. Annu. Rev. Biophys. Bioeng. 8, 385–416. (10.1146/annurev.bb.08.060179.002125) 383006

[211] Nakano T, Kyozuka K, Deguchi R 2008 Novel two-step Ca^2+^ increase and its mechanisms and functions at fertilization in oocytes of the annelidan worm *Pseudopotamilla occelata*. Dev. Growth Differ. 50, 365–379. (10.1111/j.1440-169X.2008.01022.x) 18445067

[212] Gould MC, Stephano JL, De Ortz-Barrn BJ, Prez-Quezada I. 2001 Maturation and fertilization in *Lottia gigantea* oocytes: intracellular pH, Ca^2+^, and electrophysiology. J. Exp. Zool. 290, 411–420. (10.1002/jez.1082) 11550189

[213] Miyazaki S, Igusa Y 1981 Fertilization potential in golden hamster eggs consists of recurring hyperpolarizations. Nature 290, 702–704. (10.1038/290702a0) 6894326

[214] Jaffe LA, Sharp AP, Wolf DP 1983 Absence of an electrical polyspermy block in the mouse. Dev. Biol. 96, 317–323. (10.1016/0012-1606(83)90168-9) 6832474

[215] Peres A 1987 The calcium current of mouse egg measured in physiological calcium and temperature conditions. J. Physiol. 391, 573–588. (10.1113/jphysiol.1987.sp016757) 2451013 PMC1192233

[216] Day ML, Johnson MH, Cook DI 1998 Cell cycle regulation of a T-type calcium current in early mouse embryos. Pflugers Arch. Eur. J. Physiol. 436, 834–842. (10.1007/s004240050712) 9799396

[217] Bernhardt ML, Zhang Y, Erxleben CF, Padilla-banks E, Mcdonough CE, Miao Y, Armstrong DL, Williams CJ 2015 CaV3.2T-type channels mediate Ca^2+^ entry during oocyte maturation and following fertilization. J. Cell Sci. 128, 4442–4452. (10.1242/jcs.180026) 26483387 PMC4712821

[218] Saul S, Stanisz H, Backes CS, Schwarz EC, Hoth M 2014 How ORAI and TRP channels interfere with each other: interaction models and examples from the immune system and the skin. Eur. J. Pharmacol. 739, 49–59. (10.1016/j.ejphar.2013.10.071) 24291108

[219] Birnbaumer L 2015 From GTP and G proteins to TRPC channels: a personal account. J. Mol. Med. 93, 941–953. (10.1007/s00109-015-1328-5) 26377676

[220] Xu H, Delling M, Jun JC, Clapham DE 2006 Oregano, thyme and clove-derived flavors and skin sensitizers activate specific TRP channels. Nat. Neurosci. 9, 628–635. (10.1038/nn1692) 16617338

[221] Carvacho I, Lee HC, Fissore RA, Clapham DE 2013 TRPV3 channels mediate strontium-induced mouse-egg activation. Cell Rep. 5, 1375–1386. (10.1016/j.celrep.2013.11.007) 24316078 PMC3918412

[222] Nadler MJS et al 2001 LTRPC7 is a Mg·ATP-regulated divalent cation channel required for cell viability. Nature 411, 590–595. (10.1038/35079092) 11385574

[223] Xiao E, Yang HQ, Gan YH, Duan DH, He LH, Guo Y, Wang SQ, Zhang Y 2015 Brief reports: TRPM7 senses mechanical stimulation inducing osteogenesis in human bone marrow mesenchymal stem cells. Stem Cells 33, 615–621. (10.1002/stem.1858) 25263397

[224] Runnels LW, Yue L, Clapham DE 2001 TRP-PLIK, a bifunctional protein with kinase and ion channel activities. Science 291, 1043–1047. (10.1126/science.1058519) 11161216

[225] Carvacho I, Ardestani G, Lee HC, McGarvey K, Fissore RA, Lykke-Hartmann K 2016 TRPM7-like channels are functionally expressed in oocytes and modulate post-fertilization embryo development in mouse. Sci. Rep. 6, 1–12. (10.1038/srep34236) 27681336 PMC5041074

[226] Horner VL, Wolfner MF 2008 Mechanical stimulation by osmotic and hydrostatic pressure activates *Drosophila* oocytes *in vitro* in a calcium-dependent manner. Dev. Biol. 316, 100–109. (10.1016/j.ydbio.2008.01.014) 18304524 PMC2372165

[227] Loeb J 1899 On the nature of the process of fertilization and the artificial production of normal larvæ (plutei) from the unfertilized eggs of the sea urchin. Am. J. Physiol. Content 3, 135–138. (10.1152/ajplegacy.1899.3.3.135)

[228] Loeb J 1914 Activation of the unfertilized egg by ultra-violet rays. Science 40, 680–681. (10.1126/science.40.1036.680) 17742992

[229] Lillie R 1926 The activation of starfish eggs by acids. J. Gen. Physiol. 8, 339–367. (10.1085/jgp.8.4.339) 19872205 PMC2140768

[230] Cattell W 1926 Electrical activation of the nereis egg. Science 64, 558–560. (10.1126/science.64.1666.558-a) 17835163

[231] Pincus G, Enzmann E 1936 The comparative behavior of mammalian eggs *in vivo* and *in vitro*. II. The activation of tubal eggs of the rabbit. J. Exp. Zool. 73, 195–208. (10.1084/jem.62.5.665) PMC2133299 19870440

[232] Surani MA, Barton SC, Norris ML 1984 Development of reconstituted mouse eggs suggests imprinting of the genome during gametogenesis. Nature 308, 548–550. (10.1038/308548a0) 6709062

[233] McGrath J, Solter D 1984 Completion of mouse embryogenesis requires both the maternal and paternal genomes. Cell 37, 179–183. (10.1016/0092-8674(84)90313-1) 6722870

[234] Nakada K, Mizuno J 1998 Intracellular calcium responses in bovine oocytes induced by spermatozoa and by reagents. Theriogenology 50, 269–282. (10.1016/s0093-691x(98)00135-6) 10734495

[235] Vanden Meerschaut F, Nikiforaki D, Heindryckx B, De Sutter P. 2014 Assisted oocyte activation following ICSI fertilization failure. Reprod. Biomed. Online 28, 560–571. (10.1016/j.rbmo.2014.01.008) 24656559

[236] Zhu J, Telfer EE, Fletcher J, Springbett A, Dobrinsky JR, De Sousa PA, Wilmut I. 2002 Improvement of an electrical activation protocol for porcine oocytes. Biol. Reprod. 66, 635–641. (10.1095/biolreprod66.3.635) 11870069

[237] Joshi RP, Schoenbach KH 2002 Mechanism for membrane electroporation irreversibility under high-intensity, ultrashort electrical pulse conditions. Phys. Rev. E. Stat. Nonlin. Soft Matter Phys. 66, 52901 (10.1103/PhysRevE.66.052901) 12513540

[238] Batista Napotnik T, Reberšek M, Vernier PT, Mali B, Miklavčič D 2016 Effects of high voltage nanosecond electric pulses on eucaryotic cells (*in vitro*): a systematic review. Bioelectrochemistry 110, 1–12. (10.1016/j.bioelechem.2016.02.011) 26946156

[239] Liu J, Lu Q, Liang R, Guo J, Wang K, Dong F, Wang J, Zhang J, Fang J 2019 Communicating with mouse oocytes via regulating calcium oscillation patterns by nanosecond pulsed electric fields. Phys. Rev. Appl. 11, 024001 (10.1103/PhysRevApplied.11.024001)

[240] Morgan AJ, Jacob R 1994 Ionomycin enhances Ca^2+^ influx by stimulating store-regulated cation entry and not by a direct action at the plasma membrane. Biochem. J. 300, 665–672. (10.1042/bj3000665) 8010948 PMC1138219

[241] Nikiforaki D, Vanden Meerschaut F, De Roo C, Lu Y, Ferrer-Buitrago M, De Sutter P, Heindryckx B. 2016 Effect of two assisted oocyte activation protocols used to overcome fertilization failure on the activation potential and calcium releasing pattern. Fertil. Steril. 105, 798–806. (10.1016/j.fertnstert.2015.11.007) 26632207

[242] Rickords LF, White KL 1993 Electroporation of inositol 1,4,5-triphosphate induces repetitive calcium oscillations in murine oocytes. J. Exp. Zool. 265, 178–184. (10.1002/jez.1402650209) 8423441

[243] Balakier H, Casper RF 1993 Experimentally induced parthenogenetic activation of human oocytes. Hum. Reprod. 8, 740–743. (10.1093/oxfordjournals.humrep.a138132) 8314970

[244] Ruddock NT, Machaty Z, Cabot RA, Prather RS 2001 Porcine oocyte activation: differing roles of calcium and pH. Mol. Reprod. Dev. 59, 227–234. (10.1002/mrd.1027) 11389559

[245] Presicce GA, Yang X 1994 Parthenogenetic development of bovine oocytes matured *in vitro* for 24 hr and activated by ethanol and cycloheximide. Mol. Reprod. Dev. 38, 380–385. (10.1002/mrd.1080380405) 7980946

[246] Fujinami N, Hosoi Y, Kato H, Matsumoto K, Saeki K, Iritani A 2004 Activation with ethanol improves embryo development of ICSI-derived oocytes by regulation of kinetics of MPF activity. J. Reprod. Dev. 50, 171–178. (10.1262/jrd.50.171) 15118243

[247] Wang Z, Wang W, Yu S, Xu Z 2008 Effects of different activation protocols on preimplantation development, apoptosis and ploidy of bovine parthenogenetic embryos. Anim. Reprod. Sci. 105, 292–301. (10.1016/j.anireprosci.2007.03.017) 17475421

[248] Fraser LR 1987 Strontium supports capacitation and the acrosome reaction in mouse sperm and rapidly activates mouse eggs. Gamete Res. 18, 363–374. (10.1002/mrd.1120180410) 3507382

[249] Zhang D, Pan L, Yang L-H, He X-K, Huang X-Y, Sun F-Z 2005 Strontium promotes calcium oscillations in mouse meiotic oocytes and early embryos through InsP3 receptors, and requires activation of phospholipase and the synergistic action of InsP3. Hum. Reprod. 20, 3053–3061. (10.1093/humrep/dei215) 16055456

[250] Barbosa Fernandes C, Devito LG, Martins LR, Blanco IDP, De Lima Neto JF, Tsuribe PM, Gonçalves CGP, Da Cruz Landim-Alvarenga F. 2014 Artificial activation of bovine and equine oocytes with cycloheximide, roscovitine, strontium, or 6-dimethylaminopurine in low or high calcium concentrations. Zygote 22, 387–394. (10.1017/S0967199412000627) 23340077

[251] Yamazaki W, Ferreira CR, Meo SC, Leal CLV, Meirelles FV, Garcia JM 2005 Use of strontium in the activation of bovine oocytes reconstructed by somatic cell nuclear transfer. Zygote 13, 295–302. (10.1017/S0967199405003333) 16388697

[252] Che L, Lalonde A, Bordignon V 2007 Chemical activation of parthenogenetic and nuclear transfer porcine oocytes using ionomycin and strontium chloride. Theriogenology 67, 1297–1304. (10.1016/j.theriogenology.2007.02.006) 17350088

[253] Lu Y et al 2018 Strontium fails to induce Ca^2+^ release and activation in human oocytes despite the presence of functional TRPV3 channels. Hum. Reprod. Open 2018, 1–11. (10.1093/hropen/hoy005) PMC6276696 30895246

[254] Swann K 2018 The role of Ca^2+^ in oocyte activation during *in vitro* fertilization: insights into potential therapies for rescuing failed fertilization. Biochim. Biophys. Acta—Mol. Cell Res. 1865, 1830–1837. (10.1016/j.bbamcr.2018.05.003) 29746897

[255] Swann K 1991 Thimerosal causes calcium oscillations and sensitizes calcium-induced calcium release in unfertilized hamster eggs. FEBS Lett. 278, 175–178. (10.1016/0014-5793(91)80110-O) 1991508

[256] Cheek TR, McGuinness OM, Vincent C, Moreton RB, Berridge MJ, Johnson MH 1993 Fertilisation and thimerosal stimulate similar calcium spiking patterns in mouse oocytes but by separate mechanisms. Development 119, 179–189. 8275854 10.1242/dev.119.1.179

[257] Alexandre H, Delsinne V, Goval J-J 2003 The thiol reagent, thimerosal, irreversibly inhibits meiosis reinitiation in mouse oocyte when applied during a very early and narrow temporal window: a pharmacological analysis. Mol. Reprod. Dev. 65, 454–461. (10.1002/mrd.10319) 12840819

[258] Machaty Z, Wang WH, Day BN, Prather RS 1997 Complete activation of porcine oocytes induced by the sulfhydryl reagent, thimerosal. Biol. Reprod. 57, 1123–1127. (10.1095/biolreprod57.5.1123) 9369179

[259] McDougall A, Gillot I, Whitaker M 1993 Thimerosal reveals calcium-induced calcium release in unfertilised sea urchin eggs. Zygote 1, 35–42. (10.1017/s0967199400001271) 8081800

[260] Herbert M, Gillespie J, Murdoch A 1997 Development of calcium signalling mechanisms during maturation of human oocytes. Mol. Hum. Reprod. 3, 965–973. (10.1093/molehr/3.11.965) 9433922

[261] Hinrichs K, Choi YH, Varner DD, Hartman DL 2007 Production of cloned horse foals using roscovitine-treated donor cells and activation with sperm extract and/or ionomycin. Reproduction 134, 319–325. (10.1530/REP-07-0069) 17660241

[262] Prukudom S, Perez GI, Cibelli JB, Siripattarapravat K 2019 Use of soluble sperm extract to improve cloning efficiency in zebrafish. Int. J. Dev. Biol. 63, 287–293. (10.1387/ijdb.180367ks) 31250912

[263] Ross PJ, Rodriguez RM, Iager AE, Beyhan Z, Wang K, Ragina N, Yoon SY, Fissore RA, Cibelli JB 2009 Activation of bovine somatic cell nuclear transfer embryos by PLCZ cRNA injection. Reproduction 137, 427–437. (10.1530/REP-08-0419) 19074500

[264] Yamaguchi T, Ito M, Kuroda K, Takeda S, Tanaka A 2017 The establishment of appropriate methods for egg-activation by human PLCZ1 RNA injection into human oocyte. Cell Calcium 65, 22–30. (10.1016/j.ceca.2017.03.002) 28320563

[265] Sanusi R, Yu Y, Nomikos M, Lai FA, Swann K 2015 Rescue of failed oocyte activation after ICSI in a mouse model of male factor infertility by recombinant phospholipase C*ζ*. Mol. Hum. Reprod. 21, 783–791. (10.1093/molehr/gav042) 26187950 PMC4586348

[266] Yoon SY et al 2012 Recombinant human phospholipase C zeta 1 induces intracellular calcium oscillations and oocyte activation in mouse and human oocytes. Hum. Reprod. 27, 1768–1780. (10.1093/humrep/des092) 22456923

[267] Tarín JJ, Pérez-Albalá S, Cano A 2000 Consequences on offspring of abnormal function in ageing gametes. Hum. Reprod. Update 6, 532–549. (10.1093/humupd/6.6.532) 11129687

[268] Jones KT, Whittingham DG 1996 A comparison of sperm- and IP3-induced Ca^2+^ release in activated and aging mouse oocytes. Dev. Biol. 178, 229–237. (10.1006/dbio.1996.0214) 8812125

[269] Igarashi H, Takahashi E, Hiroi M, Doi K 1997 Aging-related changes in calcium oscillations in fertilized mouse oocytes. Mol. Reprod. Dev. 48, 383–390. (10.1002/(SICI)1098-2795(199711)48:3<383::AID-MRD12>3.0.CO;2-X) 9322251

[270] Gordo AC, Rodrigues P, Kurokawa M, Jellerette T, Exley GE, Warner C, Fissore R 2002 Intracellular calcium oscillations signal apoptosis rather than activation in *in vitro* aged mouse eggs. Biol. Reprod. 66, 1828–1837. (10.1095/biolreprod66.6.1828) 12021069

[271] Takahashi T, Igarashi H, Kawagoe J, Amita M, Hara S, Kurachi H 2009 Poor embryo development in mouse oocytes aged *in vitro* is associated with impaired calcium homeostasis. Biol. Reprod. 80, 493–502. (10.1095/biolreprod.108.072017) 19038861

[272] Zhang N, Wakai T, Fissore RA 2011 Caffeine alleviates the deterioration of Ca^2+^ release mechanisms and fragmentation of *in vitro*-aged mouse eggs. Mol. Reprod. Dev. 78, 684–701. (10.1002/mrd.21366) 22095868 PMC3227013

[273] Szpila M, Walewska A, Sabat-Pośpiech D, Strączyńska P, Ishikawa T, Milewski R, Szczepańska K, Ajduk A 2019 Postovulatory ageing modifies sperm-induced Ca^2+^ oscillations in mouse oocytes through a conditions-dependent, multi-pathway mechanism. Sci. Rep. 9, 11859 (10.1038/s41598-019-48281-3) 31413272 PMC6694115

[274] Zhao S, Liu ZX, Bao ZJ, Wu Y, Wang K, Yu GM, Wang CM, Zeng SM 2015 Age-associated potency decline in bovine oocytes is delayed by blocking extracellular Ca^2+^ influx. Theriogenology 83, 1493–1501. (10.1016/j.theriogenology.2015.01.034) 25784452

[275] Hirota J, Furuichi T, Mikoshiba K 1999 Inositol 1,4,5-trisphosphate receptor type I is a substrate for caspase-3 and is cleaved during apoptosis in a caspase-3-dependent manner. J. Biol. Chem. 274, 34 433–34 437. (10.1074/jbc.274.48.34433) 10567423

[276] Verbert L et al 2008 Caspase-3-truncated type 1 inositol 1,4,5-trisphosphate receptor enhances intracellular Ca^2+^ leak and disturbs Ca^2+^ signalling. Biol. Cell 100, 39–49. (10.1042/bc20070086) 17868032 PMC2909191

[277] Gordo AC, Wu H, He CL, Fissore RA 2000 Injection of sperm cytosolic factor into mouse metaphase II oocytes induces different developmental fates according to the frequency of [Ca^2+^]_i_ oscillations and oocyte age. Biol. Reprod. 62, 1370–1379. (10.1095/biolreprod62.5.1370) 10775189

[278] Campbell K, Swann K 2006 Ca^2+^ oscillations stimulate an ATP increase during fertilization of mouse eggs. Dev. Biol. 298, 225–233. (10.1016/j.ydbio.2006.06.032) 16872595

